# Characterization of Potential Polysaccharide Utilization Systems in the Marine *Bacteroidetes Gramella Flava* JLT2011 Using a Multi-Omics Approach

**DOI:** 10.3389/fmicb.2017.00220

**Published:** 2017-02-14

**Authors:** Kai Tang, Yingfan Lin, Yu Han, Nianzhi Jiao

**Affiliations:** State Key Laboratory for Marine Environmental Science, Institute of Marine Microbes and Ecospheres, Xiamen UniversityXiamen, China

**Keywords:** glycoside hydrolases, polysaccharide utilization loci, *Bacteroidetes*, genome, microarray, proteomics, marine bacteria

## Abstract

Members of phylum *Bacteroidetes* are distributed across diverse marine niches and *Flavobacteria* is often the predominant bacterial class decomposing algae-derived polysaccharides. Here, we report the complete genome of *Gramella flava* JLT2011 (*Flavobacteria*) isolated from surface water of the southeastern Pacific. A remarkable genomic feature is that the number of glycoside hydrolase (GH) genes in the genome of *G. flava* JLT2011 is more than 2-fold higher than that of other *Gramella* species. The functional profiles of the GHs suggest extensive variation in *Gramella* species. Growth experiments revealed that *G. flava* JLT2011 has the ability to utilize a wide range of polysaccharides for growth such as xylan and homogalacturonan in pectin. Nearly half of all GH genes were located on the multi-gene polysaccharide utilization loci (PUL) or PUL-like systems in *G. flava* JLT2011. This species was also found to harbor the two xylan PULs and a pectin PUL, respectively. Gene expression data indicated that more GHs and sugar-specific outer-membrane *susC-susD* systems were found in the presence of xylan than in the presence of pectin, suggesting a different strategy for heteropolymeric xylan and homoglacturonan utilization. Multi-omics data (transcriptomics, proteomics, and metabolomics) indicated that xylan PULs and pectin PUL are respectively involved in the catabolism of their corresponding polysaccharides. This work presents a comparison of polysaccharide decomposition within a genus and expands current knowledge on the diversity and function of PULs in marine *Bacteroidetes*, thereby deepening our understanding of their ecological role in polysaccharide remineralization in the marine system.

## Introduction

Members of the *Bacteroidetes*, formerly known as the *Cytophaga-Flavobacteria-Bacteroides* phylum, are diverse and widely distributed in marine ecosystems (Glöckner et al., 1999; Kirchman, 2002; Alonso et al., 2007). Many have gliding motility as well as the capacity to degrade polymeric substances, possibly enabling them to grow on organic aggregates or algal cells using polymeric substances as carbon and energy sources (Bauer et al., 2006; McBride et al., 2009; Qin et al., 2010; Mann et al., 2013). These bacteria use their wide array of peptidases and carbohydrate-active enzymes (CAZymes), including glycoside hydrolases (GHs), carbohydrate esterases (CEs), polysaccharide lyases (PLs), and glycoside transferases (GTs) to efficiently degrade polymers such as proteins and polysaccharides (Bauer et al., 2006; Fernández-Gómez et al., 2013). Several GH genes in the genomes of *Bacteroidetes* are organized in distinct polysaccharide utilization loci (PUL), often along with PL and CE genes, and regulatory elements (Martens et al., 2011; Kabisch et al., 2014). These are also likely to be clustered with transporter systems that are involved in the binding and uptake of oligosaccharides, including susD-like oligosaccharide-binding proteins and susC-like TonB-dependent receptors (porins that mediate transport of starch oligosaccharides from the surface of the outer membrane to the periplasm for subsequent degradation; Anderson and Salyers, 1989a,b; Shipman et al., 2000). Despite the fact that PULs can be predicted based on their genomic context (Terrapon et al., 2015), recent studies have focused on the experimental validation of PULs in this group because PULs are integral to the digestion of complex carbohydrates. For example, a xyloglucan PUL, xylan PULs, and a mannan PUL were identified in *Bacteroidetes ovatus* (Larsbrink et al., 2014; Rogowski et al., 2015) and in *B. thetaiotaomicron* (Cuskin et al., 2015), respectively.

Phytoplankton contribute approximately 50% of the global primary production (Boyce et al., 2010) and bacteria can consume a substantial fraction of primary production as dissolved organic carbon (DOC) that is released by algal cells (Buchan et al., 2014). Polysaccharides constitute a large percentage of phytoplankton biomass, particulate organic matter, and DOC in the ocean (Benner et al., 1992; Engel et al., 2004). The quantity of CAZymes in phylum *Bacteroidetes* increases in the presence of phytoplankton blooms (Teeling et al., 2012). Among the members of marine *Bacteroidetes* that are associated with phytoplankton blooms, a high proportion is represented by class *Flavobacteria* (Alonso et al., 2007; Teeling et al., 2012). These are the predominant bacteria that utilize phytoplankton-derived transparent exopolymer particles in the surface coastal waters (Taylor et al., 2014). Polysaccharides are constituents of marine algae such as alginate and laminarin in brown algae, xylan in green algae and red algae, and pectin in diatoms (Kröger and Poulsen, 2008; De Jesus Raposo et al., 2014; Based et al., 2015). These algae are significant contributors to primary production in the marine environment (Nelson et al., 1995).

The breakdown of algal polysaccharides by *Bacteroidetes* is central to marine carbon cycling (Cottrell and Kirchman, 2000); however, the mechanisms underlying the action of complex polysaccharides such as xylan and pectin remain unclear. *Gramella forsetii* KT0803, which belongs to class *Flavobacteria*, is the first marine *Bacteroidetes* that was sequenced and analyzed and was found to contain a substantial number of CAZymes and peptidases, as well as a predicted preference for polymeric carbon sources (Bauer et al., 2006). The presence of a starch PUL, laminarin PUL, and alginate PUL in *G. forsetii* KT0803 has been further resolved by functional proteomics analysis (Kabisch et al., 2014). Members of genus *Gramella* occur in a variety of marine habitats, including coastal regions (*G. forsetii* KT0803) (Bauer et al., 2006), open oceans (*G. flava* JLT2011) (Liu et al., 2014), marine sediments (*G. portivictoriae* DSM23547) (Lau et al., 2005), and in phytoplankton- or animal-associated domains (sea urchin-associated *G. echinicola* DSM19838; Nedashkovskaya et al., 2005). Several more genomes of *Gramella* have subsequently been sequenced (Panschin et al., 2016), and it is now theoretically possible to compare the CAZyme features of these closely related bacterial species at the genome level.

Here, we present the complete genome of *G. flava* JLT2011, in which we find more GHs than in *G. forsetii, G. portivictoriae*, and *G. echinicola*. This species also contains a xylan PUL and mannan PUL that are absent in the other species. Although *G. flava* JLT2011 lacks an alginate PUL, it was found to harbor a pectin PUL. These species exhibited differences in the spectrum of polysaccharides that an organism can potentially degrade. Using a combination of transcriptomics, proteomics, and metabolomics, the catabolic pathway models for xylan and pectin in *G. flava* JLT2011 are proposed. This study expands current knowledge on the diversity and function of PULs in marine *Bacteroidetes*.

## Materials and methods

### Gene sequencing and bioinformatics analysis

Whole genome sequencing of *G. flava* JLT2011 was performed using a hybrid approach, combining Illumina short read data with PacBio long read data (Koren et al., 2012). The genome sequences were *de novo* assembled by the HGAP2 program in the SMRT analysis server (v2.3). Illumina pair end reads were mapped to the assembled contigs to improve the accuracy of genome sequences. The final assembled genomes were automatically annotated and analyzed via the IMG/ER platform (http://img.jgi.doe.gov). The comparison and visualization of multiple genomes was conducted using BRIG (Alikhan et al., 2011), and multiple-genome alignment was performed using Mauve (Darling et al., 2009). The amino acid sequences were then submitted to the CAZyme Annotation Toolkit (Park et al., 2010; Lombard et al., 2014; http://mothra.ornl.gov/cgi-bin/cat/cat.cgi) for sequence-based annotation, with an *E*-value of 1e-40, as well as Pfam-based annotation with an *e*-value of 0.00001. The results were then further manually checked. Peptidase genes were annotated using the MEROPS peptidases database (Rawlings and Barrett, 1999). The candidates were manually examined in terms of similarity (*E*-value cutoff 1e-10) to MEROPS proteins and the presence of all catalytic sites. The cluster sequences at the gene cluster level in NCBI GenBank were detected using a MultiGeneBlast (V. 1.1.14) (Medema et al., 2013) architecture search with predicted PULs in *Gramella* species genomes as queries. The combination of a pairwise based method and phylogeny-based methods to identify orthologous genes. The predicted orthologous genes were firstly identified using OMA (V. 1.0.6) (Roth et al., 2009). The genes were finally identified as orthologs when at least two of their phylogenetic trees were reconciled with the species trees. The phylogenetic trees of the candidate orthologous genes were reconstructed with MEGA (V. 6.0) (Tamura et al., 2013), using maximum likelihood, neighbor joining distance and maximum parsimony algorithms, and implementing a JTT plus a gamma distribution with four categories. The concatenated conserved eight single-copy genes in the phylum *Bacteroidetes* were used for the reconstruction of the maximum likelihood species tree using the RAxML program (V. 7.4.2) (Stamatakis, 2006), implementing a GTR plus GAMMA model and performing 1000 rapid bootstrap replicates.

The complete genome sequence of *G. flava* JLT2011 has been deposited in GenBank under the accession numbers CP016359 and deposited in the Joint Genome Institute IMG/ER website (http://img.jgi.doe.gov) under the genome ID 2576861820.

### Growth experiments

All chemicals were purchased from Sigma Chemical Co. (St. Louis, MO, U.S.A.), unless otherwise stated. Both *G. flava* JLT2011 and *G. forsetii* KT0803 were cultured on minimal media [2.3% (w/v) sea salts, 0.05% (w/v) yeast extract, 0.05% NH_4_Cl (w/v) and 50 mM Tris-HCl, pH 7.8], with a final concentration of 0.2% of one of the following carbon sources: D-glucose, D-xylose, D-galacturonic acid, L-arabinose, L-rhamnose, D-mannose, D-galactose, N-acetyl-D-glucosamine, D-trehalose, xylan (oat spelts), pectin (apple), pectin (citrus peel), galatcomannan (*Ceratonia siliqua*), mannan (*Saccharomyces cerevisiae*), arabinan (sugar beet), arabinogalactan (larch), laminarin (brown algae), alginate (brown algae). and chitin (shrimp shells). Cultures using only minimal media were treated as controls. Bacteria were grown using a mechanical shaker (XMTE-8112, Sukun, China) at 180 rpm and 28°C for 7 days. Cell density was determined by optical density measured at 600 nm (OD_600_) during cultivation. Cell suspensions at the exponential growth phase were pelleted by centrifugation (14,000 g, 4°C, 20 min) for further analysis. Pectin methylesterase activity was measured as previously described (Tans-kersten et al., 1998).

### Microarray analysis

Total RNA of cells at the exponential growth phase was isolated using the Ambion RiboPure-Bacteria Kit (Ambion Waltham, USA) according to the manufacturer's instructions. Total RNA was polyadenylated using the poly(A) Tailing ATP kit (Thermo Fisher Scientific, U.S.A), and then was converted to cDNA using the T7-oligo (dT) primer and CbcScript enzymes (Capitalbio, Beijing, China) for first strand synthesis of cDNA. Using an RNase H chain, short segments of RNA were cut to obtain single stranded DNA. Using the first cDNA strand as a template, the second strand cDNA was then synthesized and the primer digested using the T7 Enzyme Mix. The double-stranded cDNA was then transcribed into cRNA followed by purification using a Clean-up Kit (MN, Düren, Germany). Then, 5 μg of cRNA along with CbcScript II enzyme (Capitalbio), using Random Primers, was used for reverse transcription. The reverse transcription product was purified by using a PCR NucleoSpinExtract II Kit (MN). This reversely transcribed product was then labeled using the KLENOW enzyme. The labeled PCR product was purified using the NucleoSpinExtract II Kit (MN) and Cy3-dCTP (GE Healthcare, Marlborough, U.S.A.). Labeled DNA was dissolved in a hybridization buffer (2X GEx Hyb Buffer (HI-RPM), 25% formamide), and hybridized overnight at 45°C. Following hybridization, the product was washed in 0.2% SDS and 2 × SSC liquid for approximately 5 min at 42°C. Following this, the product was washed in a 0.2 × SSC for 5 min at room temperature. The 60-mer oligonucleotide probes were designed by CapitalBio with online software Earray (https://earray.chem.agilent.com/earray/), according to the whole genomic sequence of *G. flava* JLT2011. Custom microarrays (8 × 15 K) with the designed oligonucleotide probes were purchased from Agilent Technologies (Santa Clara, U.S.A.). The gene chips were scanned using an Agilent G2565CA Microarray Scanner (Agilent Technologies). Images from the Agilent arrays were processed using Agilent Feature (Agilent Technologies). Signal values normalized by using the method of percentile shift after raw data were input into the GeneSpring GX software (Agilent Technologies). Differentially expressed gene analysis was carried out using GeneSpring GX software and *p*-values were calculated. Microarray data details are available at the National Center for Biotechnology Information (GEO series GSE90082).

### Proteomics analysis

Cells at the exponential growth phase were centrifuged (6,600 × g at 4°C for 10 min) and extracellular proteins in the supernatant were precipitated at 4°C using 10% trichloroacetic acid. To extract the intracellular protein fractions, cells were then washed with 10 mM Tris-HCl pH 8.0, and lysed in SDT-lysis buffer (4% SDS, 100 mM DTT, 100 mM Tris-HCl pH 8.0) using a 1:10 sample to buffer ratio for 5 min at 95°C, followed by SDS-PAGE analysis. Extracellular and intracellular protein fractions were then stored at −80°C for further protein analysis. Protein in-solution digestion was performed according to the FASP procedure (Wiśniewski et al., 2009). Proteomics analysis was performed on a Q Exactive mass spectrometer coupled to an Easy nLC (Thermo Fisher Scientific, U.S.A.). The instrument was run with the peptide recognition mode enabled. The MS/MS spectra were identified using the MASCOT engine (Matrix Science, London, UK; V. 2.2) against *G. flava* JLT2011 genomes. For protein identification, the following options were used: peptide mass tolerance = 20 ppm, MS/MS tolerance = 0.1 Da, enzyme = trypsin, max missed cleavage = 2, fixed modification: carbamidomethyl (C), and variable modification: oxidation (M). All of the reported data were based on 99% confidence for protein identification as determined by false discovery rate (FDR) ≤1%.

### Identification and quantification of metabolites in *G. flava* JLT2011 by GC-TOF/MS

Cells at the exponential growth phase were collected by centrifugation (6,600 × g at 4°C for 10 min). Subsequently, re-suspended cells were washed twice with artificial sea water at 4°C. The samples were then quenched with 5 ml 80% methanol, followed by vortex-mixing and centrifugation at 10,000 × g for 20 min at −20°C. Afterwards, 800 μl of each sample and 800 μl of methanol solution were combined in 2 ml EP tubes and mixed. Samples were then centrifuged at 12,000 rpm for 15 min at 4°C to obtain the supernatant. The above steps were repeated three times. Following this, 0.4 ml of methanol:chloroform (V methanol: V chloroform = 3:1) was added, and samples were vortex mixed for 10 s. A ball mill was then used to homogenize the samples for 5 min at 55 Hz followed by sonication for 5 min, and then centrifugation for 10 min at 4°C. Subsequently, the supernatant (approximately 0.4 ml) was transferred to a new 2 ml GC/MS glass vial. We then placed 10 μl of each sample into a new 2 ml GC/MS glass vial to use as a mixed sample for quality control. The extracts were dried in a vacuum concentrator at 30°C for approximately 1.5 h, after which 60 μl of 20 mg/ml methoxymethyl amine salt (dissolved in pyridine) was added to the dried metabolites. The samples were then mixed and sealed, and incubated at 37°C overnight in an oven. Finally, 80 ml BSTFA reagent (containing 1% TMCS) was added into the mixture and reacted at 70°C for an hour.

Metabolites extracted from the quenched *G. flava* JLT2011 cells were then examined by gas chromatography-mass spectrometry (GC-MS) using an Agilent Technologies 7890 gas chromatograph system coupled to a Pegasus HT time-of-flight (TOF) mass spectrometer (LECO Coporation, U.S.A.). The system utilized a DB-5MS capillary column coated with 5% diphenyl cross-linked with 95% dimethyl polysiloxane (30 m × 250 μm inner diameter, 0.25 μm film thickness; J&W Scientific, Folsom, CA, U.S.A.). A 1 μL aliquot of the analyte was injected in splitless mode. Helium was used as the carrier gas, the front inlet purge flow was 3 mL min^−1^ and the gas flow rate through the column was 20 mL min^−1^. The initial temperature was kept at 50°C for 1 min, rose to 330°C at a rate of 20°C min^−1^, and then maintained for 1 min at 330°C. The injection, transfer line and ion source temperatures were 330, 280, and 250°C, respectively. The energy was −70 eV in electron impact mode. The mass spectrometry data were acquired in full-scan mode with the m/z range of 30–600 at a rate of 20 spectra per second after a solvent delay of 366 s. The Chroma TOF4.3X software (LECO) and LECO-Fiehn Rtx5 database were used for raw peaks extraction, data baselines filtering and calibration, peak alignment, deconvolution analysis, peak identification and integration of the peak area.

## Results and discussion

### GHs in the genome of *G. flava* JLT2011

The genome of *G. flava* JLT2011 contains a single circular chromosome of 4,007,868 base pairs (bp) in size and a GC content of 42.1%, which both are larger than that of the three *Gramella* species from distinct habitats (Table S1). The genome contains 184 genes encoding CAZymes, which is significantly higher than that of other *Gramella* species (*G. forsetii* KT0803, 118; *G. echinicola* DSM19838, 108; *G. portivictoriae* DSM23547, 119; Table S2). It also harbors 96 genes that encode GHs that represent 34 CAZyme superfamilies (Table S3), compared to *G. forsetii* KT0803 that has 42 GHs genes that were assigned to 20 superfamilies (Table S2). *G. portivictoriae* DSM23547 and *G. echinicola* DSM19838 contained only 42 and 36 GHs genes that were assigned to 19 and 16 superfamilies, respectively (Table S2). *G. flava* JLT2011 contained genes encoding 18 susCDs.

**Figure 1:**
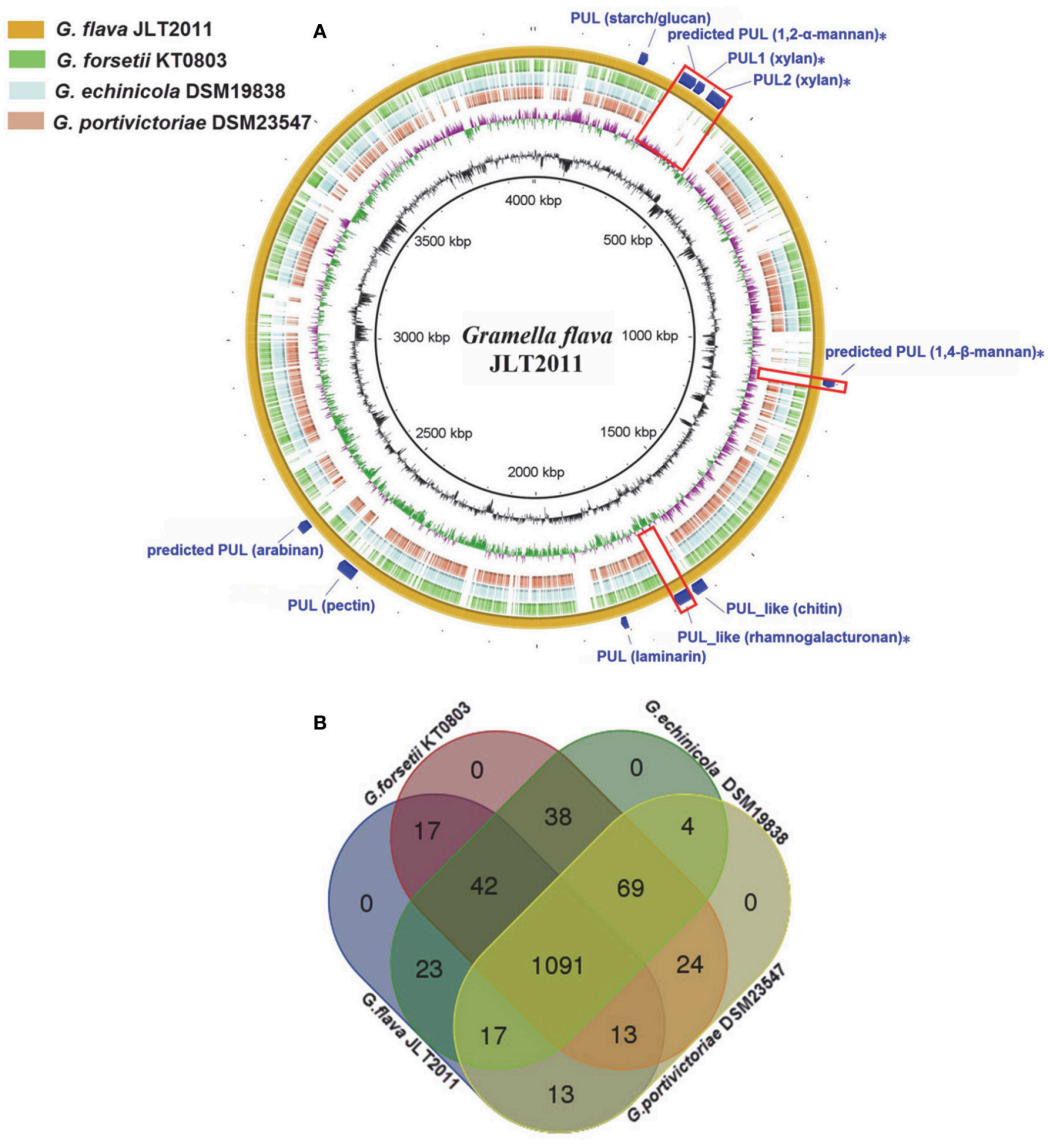
**Multiple genome comparison of ***Gramella*** species. (A)** A composite genome comparison figure was generated using BRIG after performing a BLASTn analysis of *G. flava* JLT2011 as the reference genome. Each genome mapping to the reference is represented as a colored ring, with a solid color representing >60% sequence identity. The innermost rings show GC content (black) and GC skew (purple/green) in *G. flava* JLT2011. The remaining rings indicate genome regions of *G. echinicola* (brown), *G. portivictoriae* DSM23547 (light blue), *G. forsetii* KT0803 (green), and *G. flava* JLT2011 (orange). The predicted PULs and PUL-like system gene clusters in *G. flava* JLT2011 are labeled on the outermost ring with dark blue arcs. The unique PULs in *G. flava* JLT2011 are also illustrated as red boxes and labeled with asterisks. The PUL gene cluster information is provided in Figure 3, Figures S3–S5. **(B)** Venn diagram of the intersections of orthologous genes in four genomes, where each rectangle with round corners represents one of the four genomes.

Despite the fact that *G. flava* JLT2011 and *G. forsetii* KT0803 share a large amount of homologous regions, the high number of translocations and inversions between chromosomes indicates that extensive genome rearrangements, gene gains, or gene losses have occurred (Figure S1). BLAST analysis showed that 628 genes in the *G. flava* JLT2011 genome were non-homologous to those in the other *Gramella* bacteria genomes (>30% amino acid identity; Figure 1A). A total of 1,091 orthologous genes were found among the genomes of *Gramella* species, in which only 19 GHs were determined to be orthologous (Figure 1B). Both *G. flava* JLT2011 and *G. forsetii* KT0803 shared 32 GHs homologs. However, a total of 52 genes belonging to 23 GH superfamilies in *G. flava* JLT2011 were not detected in the other three *Gramella* species. The optimal BLASTP hits of approximately two-thirds of the GHs of *G. flava* JLT2011 were aligned with non-*Gramella* bacterial species, of which most were homologous to those of bacteria from class *Flavobacteria* of phylum *Bacteroidetes* such as *Zunongwangia profunda* SM-A87 (Table S3, Figure S2). The four GH genes in *G. flava* JLT2011 that encode alpha-glucosidase, chitinase, and beta-galactosidase shared the highest similarities with those of bacteria from class *Bacteroides* (Table S3). The top hit for genes encoding endo-1,4-beta-mannosidas was determined to belong to bacteria from class *Cytophagia* (Table S3). Furthermore, a gene encoding beta-galactosidase in *G. flava* JLT2011 was best matched to the non-*Bacteroidetes* bacterium *Verrucomicrobiae* (Table S3).

### Comparative analysis of GHs and peptidases in marine microbes

The GH and peptidase genes were found to be distributed across a variety of divergent bacterial groups, including *Proteobacteria, Bacteroidetes, Cyanobacteria, Actinobacteira, Fimicutes, Planctomycetes*, and *Thermotogae* (Tables S2, S5). The numbers of GHs and peptidases in the marine *Bacteroidetes* ranged from 13 to 135 per genome (median: 46) and from 76 to131 (median: 98), respectively (Tables S2, S4). The median frequency of GHs and peptidases in the marine members of phylum *Bacteroidetes* was 12 GHs per Mbp and 24 peptidases per Mbp, accounting for 1.4 and 2.6% of the total open reading frames, respectively. This was higher than that of the other taxa that contained more than three genomes (Table S2).

**Figure 2:**
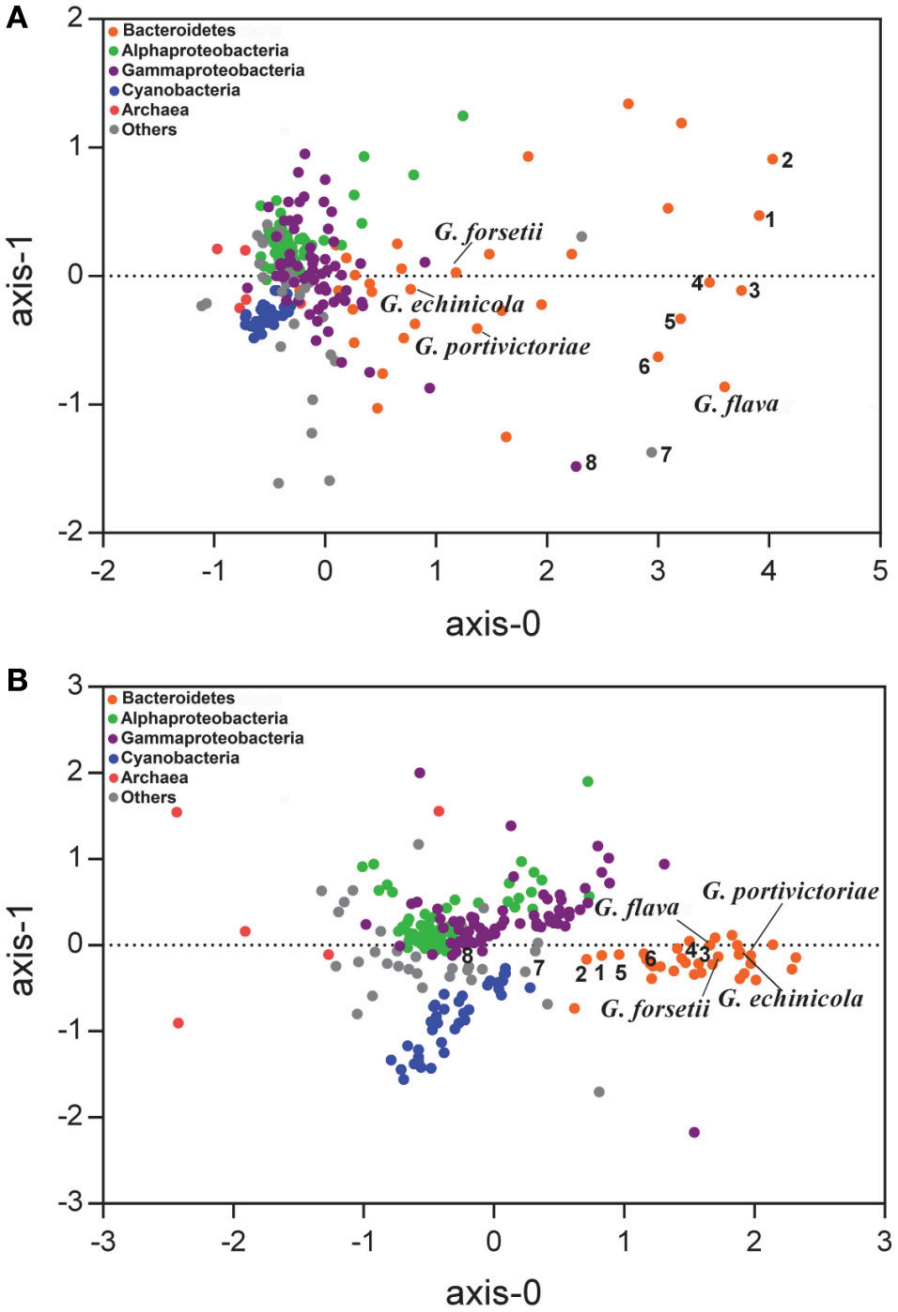
**Nonmetric multidimensional scaling analysis based on Bray-Curtis similarity matrix was calculated from the frequency of each GH (A)** and peptidase **(B)** in marine bacteria. A total of 249 marine bacteria genomes were included in the analysis and their GHs and peptidases of each protein superfamily were standardized by the genome size (see details on Tables S2, S4). The bacterial lineages are highlighted in colors. 1. *Echinicola vietnamensis* DSM17526; 2. *Echinicola pacifica* DSM19836; 3. *Zunongwangia profunda* SM-A78; 4. *Leeuwenhoekiella blandensis* MED217; 5. *Pedobacter* sp. BAL39; 6. *Leeuwenhoekiella* sp. Hel_I_48; 7. *Verrucomicrobiae bacterium* DG1235; 8. *Saccharophagus degradans* 2-40.

The GH functional profile in phylum *Bacteroidetes* was distinguishable from most of the other marine bacteria (Figure 2A). The *G. flava* JLT2011 genome appears to have a high frequency of GHs (>20 genes per Mbp), which is similar to other *Bacteroidetes*, including *Z. profunda* SM-A87 from deep-sea sediments (Qin et al., 2010), *Echinicola vietnamensis* DSM17526 from a lagoon of Nha Trang Bay, South China Sea (Nedashkovskaya et al., 2007), and *Leeuwenhoekiella blandensis* MED217 from the open ocean (Pinhassi et al., 2006; Figure 2A). Moreover, *Echinicola pacifica* DSM19836 contained four times as many GHs as that in *G. echinicola* DSM19838, both of which have been found in sea urchins (Nedashkovskaya et al., 2005, 2006; Table S2). These results indicate that bacteria containing abundant GHs could be found across diverse environments. Several non-*Bacteroidetes* species also harbored abundant GHs, including *Verrucomicrobiae* bacterium DG1235 and *Saccharophagus degradans* 2–40, which are efficient degraders of numerous complex polysaccharides in the marine environment (Figure 2A; Hutcheson et al., 2011; Martinez-Garcia et al., 2012).

The functional profiles of GHs in *G. flava* JLT2011 were significantly distinguishable from those of the other three *Gramella* species (Figure 2A). Furthermore, *G. flava* contained more GHs that were associated with xylan hydrolysis (GH10, GH30, GH67, and GH115 CAZyme superfamilies) than the other three *Gramella* species (Table S3). Mannan hydrolyzing GHs (GH26, GH76, GH92, and GH125) were only found in *G. flava* JLT2011 (Table S3). Pectin hydrolyzing GHs were identified in *G. flava* JLT2011 and *G. echinicola* DSM19838 (GH28, GH88, and GH105) (Table S3). While *G. forsetii* KT0803 contained seven genes that encode PLs, including alginate lyase, only two predicted pectin lyases were present in *G. flava* JLT2011 (Table S2). Differences in CAZyme distribution among the four *Gramella* species suggest variations in the genomic potential for polysaccharide degradation. In contrast, their peptidase profiles exhibited little variation (Figure 2B). Notably, *G. flava* JLT2011 contained 97 peptidases that were distributed across 39 superfamilies, whereas *G. forsetii* KT0803 comprised 102 peptidases that represented 42 superfamilies (Table S4).

### Xylan and pectin PULs in the genome of *G. flava* JLT2011

**Figure 3:**
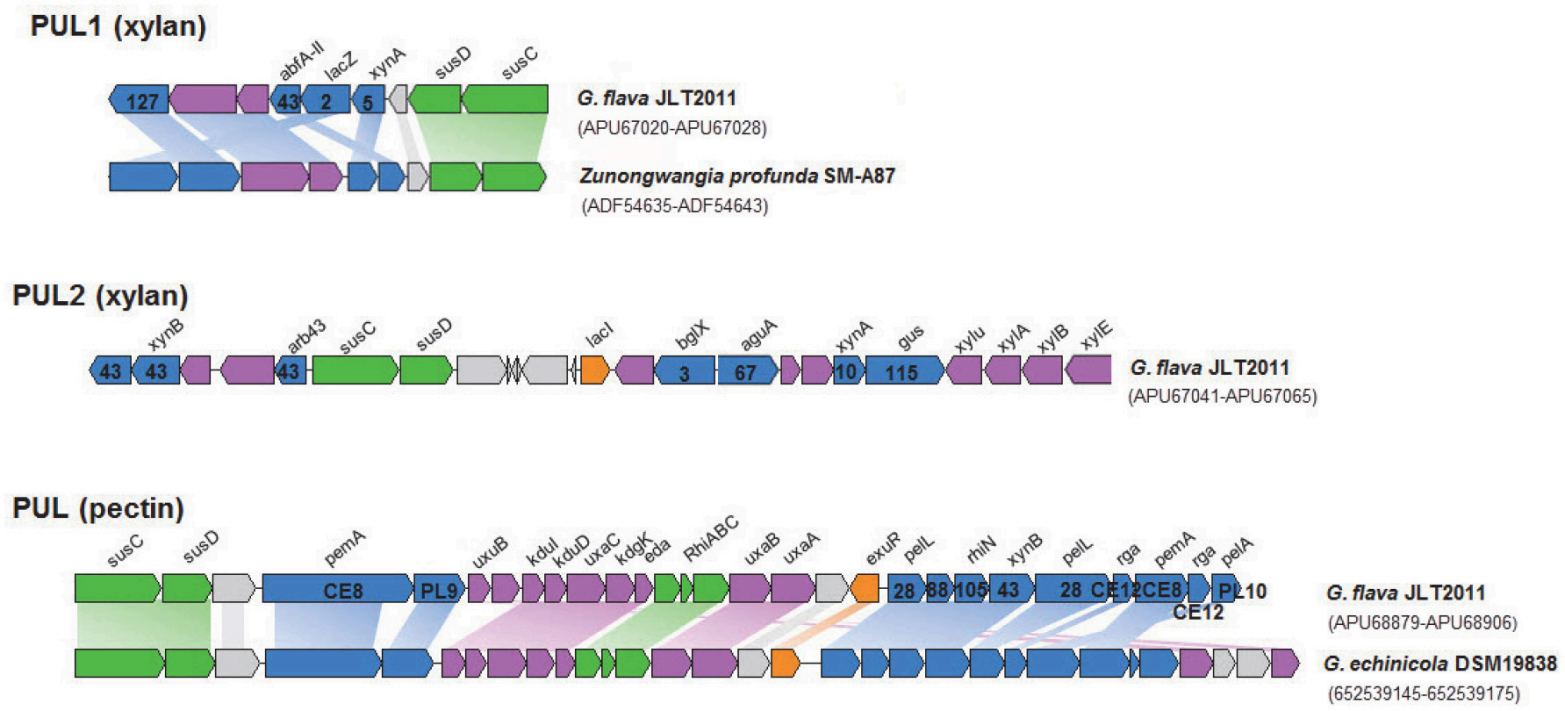
**Genetic organization of the two xylanolytic and one pectinolytic operons of ***G. flava*** JLT2011**. The range of Genbank accession numbers of sequences of the gene cluster in the genome is shown in a bracket. The functions of the proteins are color-coded: blue, CAZymes; green, membrane proteins involved in binding/transport; purple, other enzymes; orange, putative regulation factor; gray, unknown function. Homologous genes are connected by colored bars between two PULs. The CAZymes superfamily classification is listed on a gene. **xylan PUL1&2:** *xynA*, Endo-1,4-beta-xylanase; *xynB*, Beta-xylosidase; *abfA-II*, Alpha-L-arabinofuranosidase; *lacZ*, Beta-galactosidase; *arb43*, Alpha-N-arabinofuranosidase; *bglX*, Beta-glucosidase; *aguA*, Alpha-glucuronidase; *xylu*, Acetyl xylan esterase; *xylA*, Xylose isomerase; *xylB*, Xylulose kinase; *susC*, TonB-dependent receptor; *susD*, Carbohydrate-binding protein; *xylE*, Xylose transporter; *lacI*, LacI family transcriptional regulator; **pectin PUL:** *pemA*, Pectinesterase; *pelL*, Endopolygalacturonase; PL9, Pectate lyase; *pelA*, Pectate lyase; *rhiN*, Rhamnogalacturonides degradation protein; *uxuB*, D-mannonate oxidoreductase; *kduI*, 4-deoxy-L-threo-5-hexosulose-uronate ketol-isomerase; *kduD*, 2-deoxy-D-gluconate 3-dehydrogenase; *uxaC*, Uronate isomerase; *kdgK*, 2-dehydro-3-deoxygluconate kinase; *eda*, 2-dehydro-3-deoxyphosphogluconate aldolase; *uxaB*, Altronate oxidoreductase; uxaA, Altronate hydrolase; *rga*, Rhamnogalacturonan acetylesterase; *susC*, TonB-dependent receptor; *susD*, carbohydrate-binding protein; *RhiABC*, Predicted rhamnogalacturonide-specific TRAP-type transporter; *exuR*, Hexuronate utilization operon transcriptional repressor.

The PUL and PUL-like systems are variously distributed in the four *Gramella* strains (Figure 1A). Nearly half of all GHs and all *susC-susD* systems were distributed in PULs and PUL-like systems in *G. flava* JLT2011 (Figure 3, Figures S3–S5). *G. flava* JLT2011 can utilize xylan and its corresponding monosaccharide D-xylose, whereas *G. forsetii* KT0803 could not (Figure S6). *G. flava* JLT2011 was found to contain two xylan PULs that were absent in *G. forsetii* KT0803 (Figure 3). Two xylan PULs in *G. flava* JLT2011 were distinct from those in *Bacteroidetes ovatus* (Rogowski et al., 2015). However, the major xylanolytic enzymes were found in the xylan PULs of *G. flava* JLT2011, including genes that encode *xynA*-encoding xylanases (GH5 and GH10) and *xynB*-encoding beta-xylosidase (GH43) (Rogowski et al., 2015; Figure 3). Two xylan PULs both contained genes that encode a susC-susD system. Genes encoding acetyl xylan esterase (*xylU*), xylulokinase (*xylB*), xylose isomerase (*xylA*), and xylose transporters (*xylE*) were located downstream of a PUL and are probably involved in xylose utilization (Figure 3). Xylans are heteropolymers consisting principally of xylose and a small amount of arabinose, as well as traces of glucuronic acid, galactose, rhamnose, galacturonic acid, and other residues (Bastawde, 1992). The GHs that are potentially involved in the neutral sugars that are linked to the xylan main chain were found in two xylan PULs, which include alpha-N-arabinofurnosidases (*abfA-II* and *arb43*, GH43) and alpha-glucuronidases (*aguA*, GH67) (Rogowski et al., 2015; Figure 3).

Both *G. flava* JLT2011 and *G. forsetii* KT0803 can utilize pectin (from apple and citrus, respectively), and their corresponding monosaccharide, D-galacturonic acid (Figure S6, Table S5). *G. flava* JLT2011 contained a PUL that was predicted to encode for enzymes that are involved in the degradation of pectin, including two pectate lyases (*pelL* and *pelA*), two pectin methylesterases (*pemA*), and two polygalacturonases (*pelL*) (Figure 3). These enzymes cleave pectin into small oligogalacturonates. The PUL contained other genes that are involved in the catabolism of pectin such as genes encoding rhamnogalacturonides degradation proteins (*rhiN*, GH88), two rhamnogalacturonan acetylesterases, a *susC-susD* system, and a predicted transporter system for rhamnogalacturonide (*RhiABC*), and a set of genes associated with hexuronate metabolism (Figure 3). A similar PUL was identified in *G. echinicola* DSM19838 (Figure 3). *Alishewanella* (Jung and Park, 2013) and *Alteromonas* species within *Gammaproteobacteria* contained the majority of genes in a pectin PUL found in *G. flava* JLT2011. On the other hand, *G. forsetii* KT0803 lacked a pectin PUL, although it harbored a gene that encodes a pectin degradation protein, as well as other genes (including *MFS, kdgK*, and *kdgF*) located downstream of the alginate PUL locus that were possibly involved in pectin degradation (Figure S3; Bauer et al., 2006). However, the exact enzymatic mechanism of pectin for *G. forsetii* KT0803 need further experiments. Pectin methylesterase activity, which is responsible for the removal of methyl-esters from moderately methylated apple pectin, was observed when *G. flava* cells were grown on apple pectin (Figure S7). In contrast, no pectin methylesterase activity on citrus pectin was detected in *G. flava* JLT2011 because citrus pectin has a very low capability for methyl esterification. Furthermore, the absence of the *pemA* gene in *G. forsetii* KT0803 did not appear to completely prevent it from utilizing apple pectin for growth.

**Figure 4:**
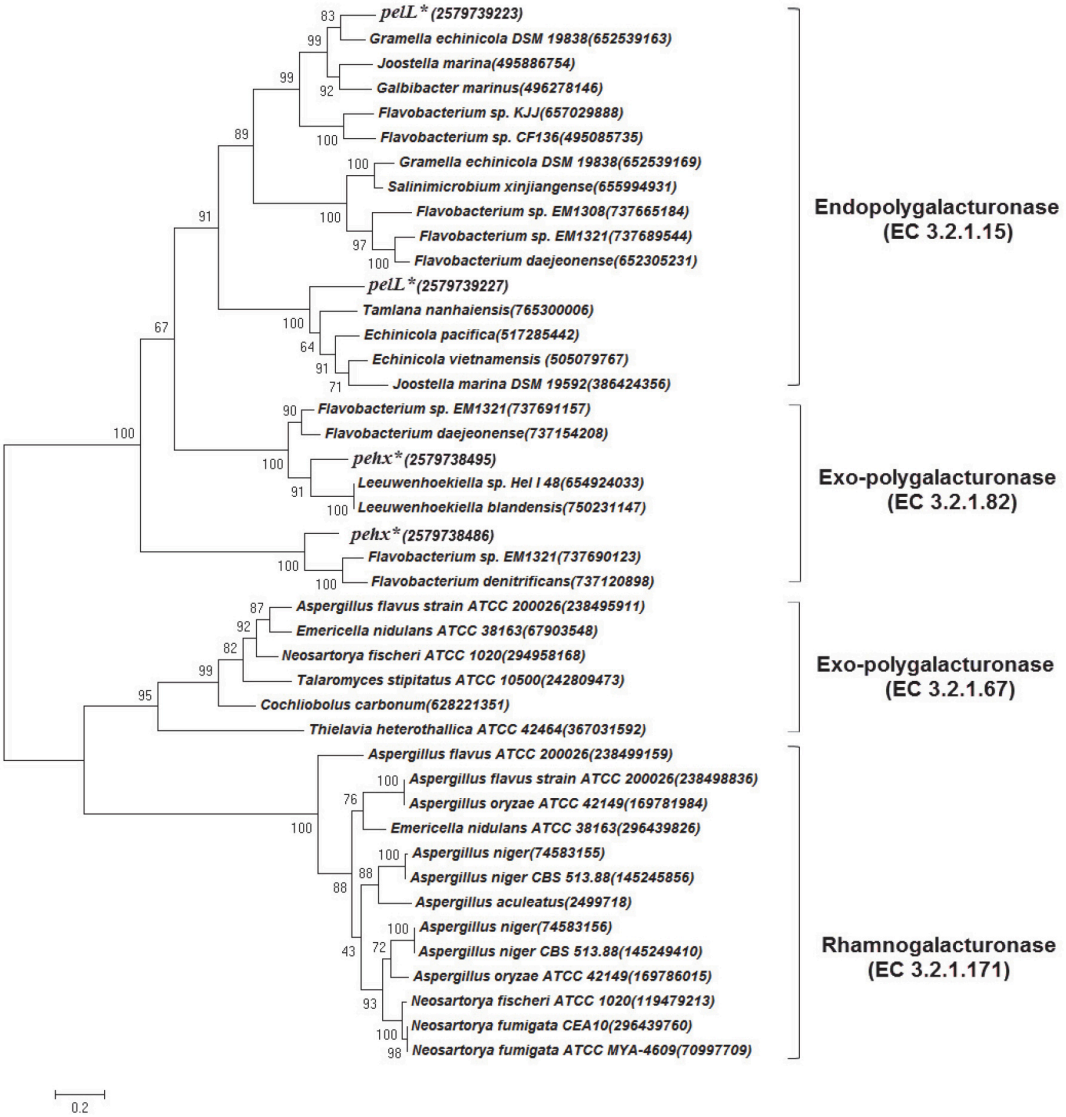
**Phylogenetic tree based on polygalacturonase amino acid sequence comparisons constructed using the maximum-likelihood method in MEGA6**. The pehX and pelL genes in *G. flava* JLT2011 are marked by asterisks and IMG/ER accession numbers are given in the bracket. The classes of polygalacturonase are listed on the right. The Genebank accession number for each reference sequence is given in the bracket. Bootstrap percentages are shown on each branch (1,000 replications). The scale bar indicates 20% sequence divergence.

Apple and citrus pectins are mainly composed of D-galacturonic acid-forming homogalacturonans. The backbone of apple pectin also contains a small amount of α-1,2-rhamnose that alternates with α-1,4-D-galacturonate (ramnogalacturonan; Wu and Mort, 2014). Other sugars, including galactose, rhamnose, glucose, arabinose, xylose, and mannose, were also detected in apple pectin (Wu and Mort, 2014). *G. flava* JLT2011 is capable of utilizing L-rhamnose as a carbon source for growth (Figure S6) due to the presence of complete L-rhamnose utilization genes (*rha*) in the genome (Figure S5). These genes were clustered with pectin lyase and GHs to form a predicted PUL-like system for rhamnogalacturonan utilization (Figure S5). Polygalacturonases act on the homoglacturonan chains of the smooth region of pectin, whereas rhamnogalacturonases digest the rhamnogalacturonan chain of the hair region of pectin (Ochiai et al., 2007). Phylogenetic analysis indicated that four genes encoding polygalacturonases in *G. flava* JL2011 functionally belonged to the endopoylygalacturonase or exo-polygalacturonase family, but were distinct from rhamnogalacturonases (Figure 4). The alternative enzymatic route for rhamnogalacturonan degradation includes rhamnogalacturonan lyase (PL4 or PL11) and rhamnogalacturonan hydrolase (GH88 and GH105) (Ochiai et al., 2007). *G. flava* JL2011 contained five rhamnogalacturonan hydrolases but lacked rhamnogalacturonan lyase. These results suggest that a PUL-like system in *G. flava* JLT2011 does not appear to function in rhamnogalacturonan hydrolysis. Both *G. flava* JLT2011 and *G. forsetii* KT0803 could only utilize the homogalacturonan in apple pectin due to the lack of rhamnogalacturonan hydrolyase or rhamnogalacturonan lyase.

Both *G. flava* JLT2011 and *G. forsetii* KT0803 harbored other PULs (Figures S3–S5); therefore, these are capable of utilizing trehalose, laminarin, starch, arabinan, arabinogalactan, galactomannan, and their corresponding monosaccharides (D-glucose, D-galactose, D-galacturonic acid, and L-arabinose; Figure S6, Table S5). These polysaccharides and corresponding monosaccharides have frequently been found in various marine algae that function in storage, or act as a cell wall constituent or as extracellular polysaccharides (Kröger and Poulsen, 2008; De Jesus Raposo et al., 2014; Based et al., 2015). Potential PUL identification and carbon utilization experiments revealed that *Gramella* species can degrade a wide range of algal polysaccharides, thus indicating a strong specialization toward an “opportunitrophic” lifestyle in the marine environment.

### Reconstruction of xylan and pectin catabolic pathways based on transcriptomics, proteomics, and metabolomics data

**Figure 5:**
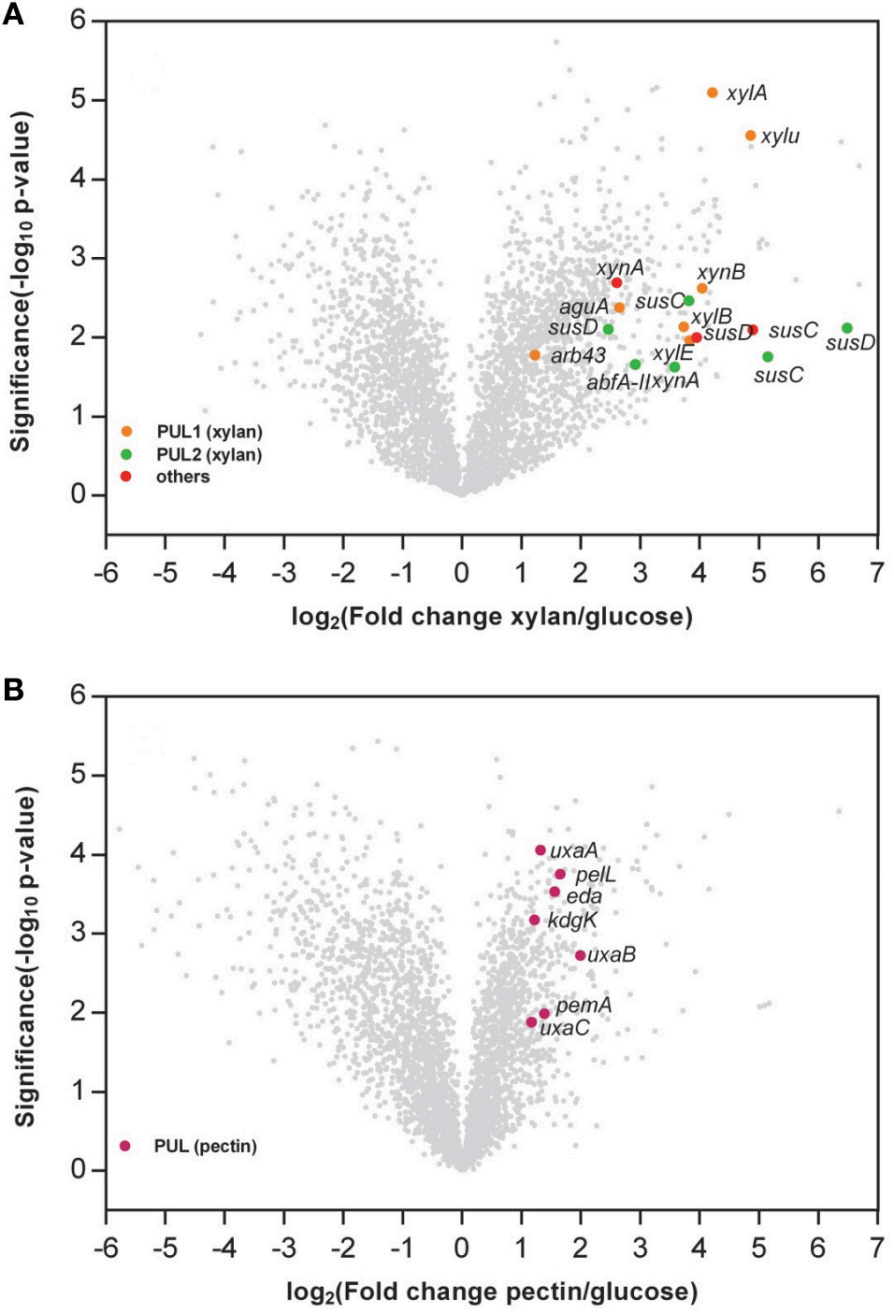
**Volcano plot comparison of the gene expression between xylan and glucose (A)** and pectin and glucose **(B)**. The x axis indicates the differential expression profiles, plotting the fold-induction ratios in a log-2 scale. The y axis indicates the statistical significance of the difference in expression (*p*-value from a *t*-test) in a log10 scale. Genes within the xylan and pectin PULs are shown in colors (fold change > 2, or < 0.5; *p* < 0.05). **(A)** *xynA*, Endo-1,4-beta-xylanase; *xynB*, Beta-xylosidase; *abfA-II*, Alpha-L-arabinofuranosidase; *arb43*, Alpha-N-arabinofuranosidase; *aguA*, Alpha-glucuronidase; *xylu*, Acetyl xylan esterase; *xylA*, Xylose isomerase; *xylB*, Xylulose kinase; *susC*, TonB-dependent receptor; *susD*, carbohydrate-binding protein; *xylE*, Xylose transporter. **(B)** *pemA*, Pectinesterase; *pelL*, Endopolygalacturonase; *uxaC*, Uronate isomerase; *kdgK*, 2-dehydro-3-deoxygluconate kinase; *eda*, 2-dehydro-3-deoxyphosphogluconate aldolase; *uxaB*, Altronate oxidoreductase; *uxaA*, Altronate hydrolase; *susC*, TonB-dependent receptor; *susD*, carbohydrate-binding protein.

Global gene expression patterns for *G. flava* JLT2011 at the exponential growth phase in the presence of glucose, xylan, and pectin were assayed using microarrays (Figure 5). Overall, compared to the glucose treatment condition, a total of 1,234 and 951 genes were differentially expressed under xylan and pectin treatment conditions, respectively (fold change >2 or < 0.5; *p* < 0.05) (Tables S6, S7). In the presence of xylan, some genes in two PULs for xylan showed increased transcript levels compared to glucose, and the induction levels varied between 2.3 and 89.6 when relative to that in glucose, including the xylan main chain degradation genes (*xynA* and *xynB*), whereas the side chain sugar degradation genes (*arb43, abfA-II*, and *aguA*), and xylose metabolism genes (*xylU, xylA, xylB*, and *xylE*) were also up-regulated (Figure 5A). Two *susC-susD* systems in xylan PULs were up-regulated, in which induction levels were up to 35.5 and 89.6 for *susC* and *susD* (Table S6), respectively, thereby suggesting that these are involved in the uptake of oligomeric xylan. In the presence of pectin, the transcription of endopolygalacturonase (*pelL*) and hexuronate metabolism (*kdgK, uxaA, uxaB, uxaC*, and *eda*) in the pectin PUL were significantly enhanced compared to glucose (Figure 5B). However, a transcription of *susC-susD* system in the pectin PUL was not significantly up-regulated, and the transcripts of *RhiABC* genes were also significantly down-regulated compared to glucose (Table S7), suggesting that these are not involved in pectin degradation. The transcripts of genes in a rhamnogalacturonan PUL-like system did not significantly change in the presence of pectin, thus supporting the notion that *G. flava* JLT2011 does not utilize heteropolymeric rhamnogalacturonans in apple pectin.

**Figure 6:**
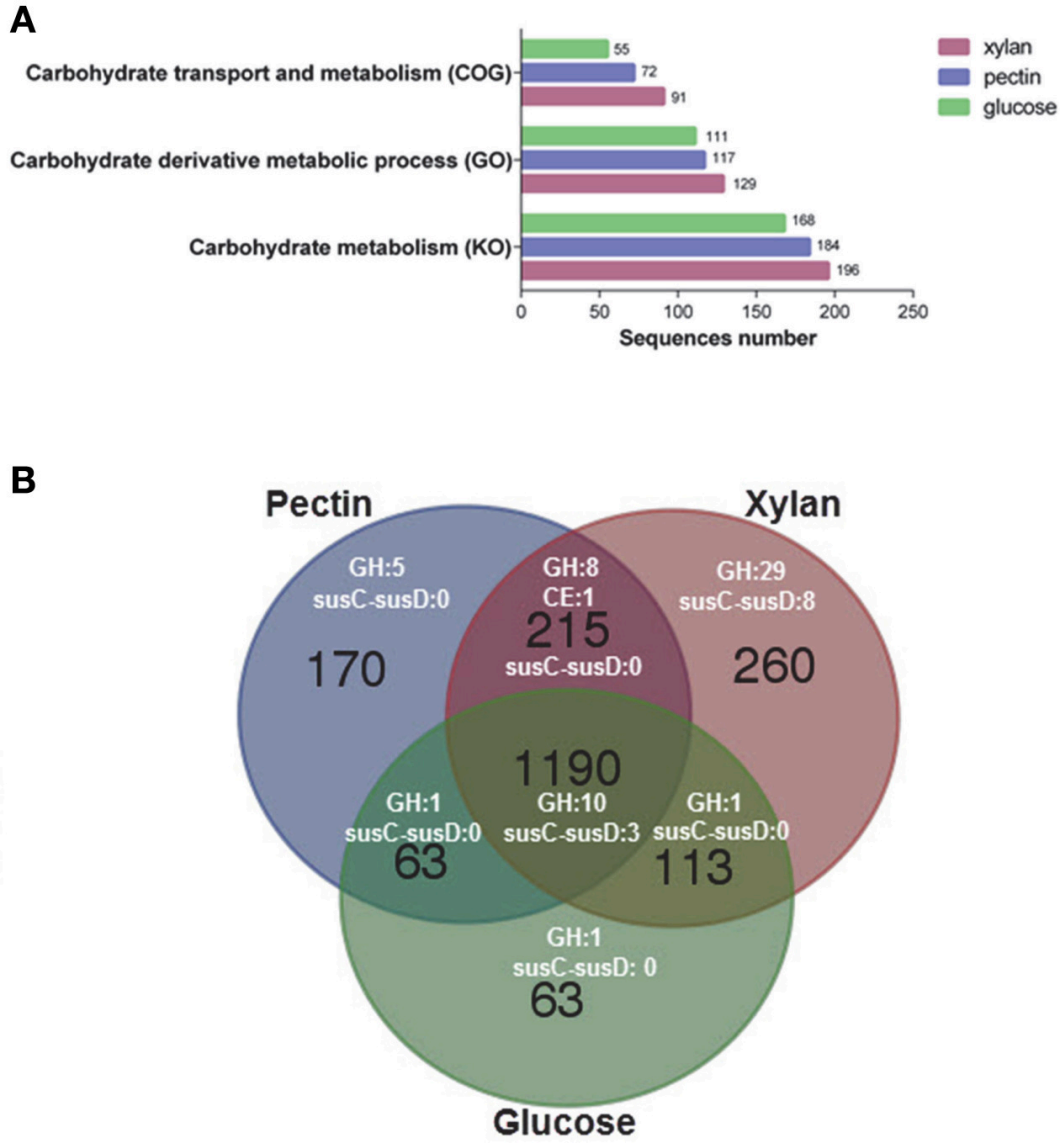
**Functional analysis of the proteome of ***G. flava*** JLT2011 grown on xylan, pectin, and glucose. (A)** Hits of identified proteins associated with carbohydrate metabolism classified by cluster of orthologous groups (COG), gene ontology (GO) and Kyoto encyclopedia of genes and genomes (KO), respectively. **(B)** Venn diagram of counts of the detected CAZymes and susC-susD systems under different treatments with xylan, pectin and glucose. GH, glycoside hydrolase; CE, carbohydrate esterase.

LC-MS/MS analysis identified a total of 1,778, 1,638, and 1,429 proteins at the exponential growth phase in the presence of xylan, pectin, and glucose, respectively (Table S8). A total of 1,190 proteins were detected among all samples, representing 33.7% of the protein-coding genes in *G. flava*. JLT2011. A higher number of proteins were associated with carbohydrate metabolism in cells cultured with xylan or pectin compared to those cultured with glucose (Figure 6A). The xylan degradation enzymes (XynA and XynB) and the endopolygalacturonases (PelL) were detected in the presence of xylan and pectin, respectively, which coincided with the results of microarray analysis. However, the SusC-susD system and RhiABC transporter in the pectin PUL were not detected, indicating that these were not involved in the transport of pectin degradation products. However, the GntP transporter was detected (Table S8) and is reported to have a high specificity for importing D-galacturonate (Suvorova et al., 2011). All *Gramella* species have the complete glycolysis and uncompleted Entner–Duodoroff and pentose phosphate pathways due to the presence of a gene that encodes glucose-6-phosphaste (P)-1-dehydrogenase. However, transketolase and transaldolase were detected in the proteomic samples (Table S8), which could convert xylose-5P into fructose-6P and glyceraldehyde-3P, which subsequently enter the glycolysis cycle. Gliding motility-related proteins, which possibly play a role in the digestion of biopolymers, were detected in the presence of xylan and pectin (Table S8).

Comparative analysis of the transcriptomics and proteomics data shows a good consistency for the PUL genes at the transcription level. All significantly up-regulated gene transcripts of xylan PULs and pectin PULs were detected at the protein level of the bacterial exponential growth phase (Table S8). Compared to glucose, a total of 25 GH and seven *susC-susD* system transcripts were up-regulated in the presence of xylan (Table S6), whereas only six GHs and a *susC-susD* system were up-regulated in the presence of pectin (Table S7). A total of 48, 24, and 13 GHs at the protein level were detected in the presence of xylan, pectin, and glucose, respectively (Figure 6B; Table S8). In addition, a total of 29 GH and eight SusC-SusD system proteins were only detected in the presence of xylan (Figure 6B). In contrast, only three SusC-SusD systems were detected in the presence of pectin and glucose (Figure 6B). Compared to glucose, the up-regulated transcript of *xynA* and its adjacent *susC-susD* system, *xynB*, were found in non-xylan PUL (Figure 5A), which possibly accelerates the degradation of the main chain of xylan. Moreover, other up-regulated transcripts of GHs, including beta-galactosidase and alpha-glucosidase, allows sufficient hydrolysis of various sugar residues of xylan (Table S6). These up-regulated transcripts in the presence of xylan are also detected at the protein level (Table S8). These results suggest that *G. flava* JLT2011 exhibited contrasting gene expression patterns on GHs and susC-susD systems in response to heteropolymeric xylan and homopolymeric pectin utilization, respectively. It was noted that some GH genes were shown differentially expressed revealed by transcriptomics in barely, while their corresponding proteins were not detected in the proteomic data possibly due to reasons such as gene expression and the limitation of techniques.

GC-MS analysis of bacterial metabolites showed that the highest concentration of xylose was observed in xylan-grown cells, whereas the highest concentration of D-galacturonic acid was detected in pectin-grown cells (Table 1). These are degradation products from xylan and pectin, respectively. The metabolites of pectin-grown cells that are involved in glycolysis (glucose-6-phosphate and fructose-6-phosphate) and the citric acid cycle cycle (citric acid, succinic acid, and fumaric acid) were down-regulated compared to that in the glucose-grown and xylan-grown cells (Table 1), indicating an decrease in the rate of glycolysis and the citric acid cycle.

**Table 1:** **Relative fold-change in selected metabolites in ***G. flava*** JLT2011 grown in different conditions (***t***-test, ***p*** < 0.05)**.

**Metabolite**	**Similarity^a^**	**R.T.^b^**	**Mass**	**log_2_ fold change (xylan/glucose)^c^**	**log_2_ fold change (pectin/glucose)^c^**
Xylose	925.89	15.07	103	2.89	0.94
D-galacturonic acid	752.50	17.96	160	0.87	1.42
Glucose	963.25	17.57	160	−0.64	−2.68
Glucose-6-phosphate	726.53	21.47	73	−0.01	−2.40
Fructose-6-phosphate	541.29	21.35	315	0.38	−2.80
Pyruvic acid	484.29	8.84	174	0.32	−1.43
Citric acid	826.63	16.77	273	−0.03	−1.50
Succinic acid	948.70	10.78	147	0.17	−0.90
Fumaric acid	927.40	11.28	245	1.20	−0.78

a *Similarity, the LECO/Fiehn Metabolomics Library was used to identify the compounds. It generates a similarity value for the compound identification accuracy. When the similarity is >700, the process of metabolite identification is reliable. When the similarity is between 200 and 700, the putative compound name is annotated. When the similarity is < 200, the library will use only “analyte” for the compound name*.

b *R.T., retention time of gas chromatography*.

c *Fold-change values using the ratios of the mean of standardized peak intensities between two groups of samples*.

**Figure 7:**
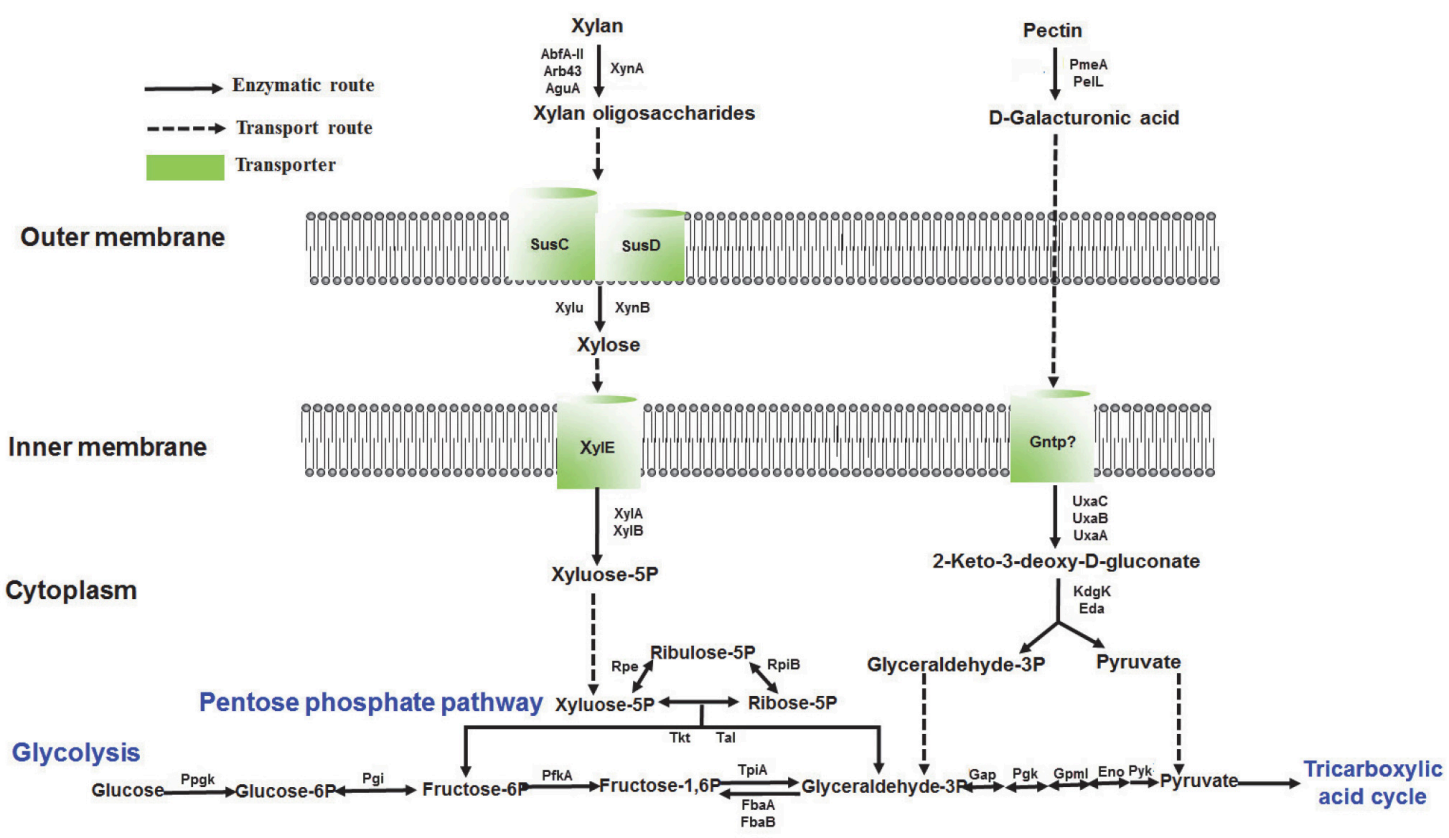
**Reconstructed pathways of xylan and pectin utilization in ***G. flava*** JLT2011 based on multi-omics data**. All labeled genes were expressed at the proteomic level. Xylan utilization pathway: XynA, Endo-1,4-beta-xylanase; XynB, Beta-xylosidase; AbfA-II, Alpha-L-arabinofuranosidase; Arb43, Alpha-N-arabinofuranosidase; AguA, Alpha-glucuronidase; SusC, TonB-dependent receptor; SusD, Carbohydrate-binding protein; Xylu, Acetyl xylan esterase; XylE, Xylose transporter; XylA, Xylose isomerase; XylB, Xylulose kinase. Pectin utilization pathway: PemA, Pectinesterase; PelL, Pectate lyase; PehX, Exo-poly-alpha-D-galacturonosidase; UxaC, Uronate isomerase; UxaB, Altronate oxidoreductase; UxaA, Altronate hydrolase; GntP, Gluconate transporter; KdgK, 2-dehydro-3-deoxygluconate kinase; Eda, 2-dehydro-3-deoxyphosphogluconate aldolase. Pentose phosphate pathway: Rpe, Ribulose-phosphate 3-epimerase; RpiB, Ribose 5-phosphate isomerase B; Tkt, Transketolase; Tal, Transaldolase. Glycolysis: PpgK, Polyphosphate glucokinase; Pgi, Glucose-6-phosphate isomerase; PfkA, 6-phosphofructokinase; TtpiA, Triosephosphate isomerase; FbaA, Fructose-bisphosphate aldolase class II; FbaB, Fructose-bisphosphate aldolase class I; Gap, NAD-dependent glyceraldehyde-3-phosphate dehydrogenase; Pgk, Phosphoglycerate kinase; GpmI, 2,3-bisphosphoglycerate-independent phosphoglycerate mutase; Eno, Enolase; Pyk, Pyruvate kinase.

The integrated multi-omics data of this study reveal that PULs play a vital role in catabolic xylan and pectin pathways and we hereby propose a xylan and pectin utilization pathway as follows. The enzyme XynA hydrolyzes the main chain of xylan into oligomeric polymers that are subsequently transported into cells by the SusC-SusD system of *G. flava* JLT2011 (Figure 7). The enzymes XynB and Xylu convert xylan oligosaccharides into xylose, which then enter the cell and move into the cytoplasm through the mediation of XylE (Figure 7). The enzymes XylA and XylB yield the final product, xylose-5P, which is then directed into the pentose phosphate pathway (Figure 7). The enzymes Arb43, AbfA-II, and AguA might be involved in hydrolysis of the side chain of xylan (Figure 7). The homogalacturonan in pectin is degraded into D-galacturonic acid via the demethylating enzyme PemA, and PelL is used in pectin hydrolysis in *G. flava* JLT2011 (Figure 7). D-galacturonic acid is further utilized by the consequent actions of five hexuronate metabolism enzymes (UxaC, UxaB, UxaA, KdgK, and Eda), converting D-galacturonic acid into pyruvate and D-glyceraldehyde-3-phosphate, which subsequently enter the glycolytic pathway (Figure 7). Further biochemical analysis of the proteins involved in xylan and pectin utilization is necessary to fully understand the systems and validate the proposed pathway.

In summary, this study has shown that the members of *Gramella* spp. have different potentials in hydrolyzing polysaccharides based on their differences in GHs and PUL genes. Genes encoding GHs and PULs in *G. flava* JLT2011 could function in the degradation of polysaccharides; however, these have distinct gene expression patterns in response to xylan and pectin, respectively. Our multi-omics studies have allowed us to functionally characterize GHs and PULs in a bacterial model that belongs phylum *Bacteroidetes*, thereby facilitating in better understanding their ecological role in algal polysaccharide decomposition in relation to marine carbon cycling.

## Author contributions

KT and NJ conceived and designed the experiments; KT, YL, and YH carried out the experiments and analyzed the data. All of the authors assisted in writing the manuscript, discussed the results and commented on the manuscript.

## Funding

This work was supported by the National Natural Science Foundation of China project (41276131), the National Key Research and Development Program of China (2016YFA0601100 & 2013CB955700), the National Program on Global Change and Air-Sea Interaction (GASI-03-01-02-05), the Natural Science Foundation of Fujian Province (2014J01164).

### Conflict of interest statement

The authors declare that the research was conducted in the absence of any commercial or financial relationships that could be construed as a potential conflict of interest.
