# Supplementary material for: Characterization of Potential Polysaccharide Utilization Systems in the Marine Bacteroidetes Gramella Flava JLT2011 Using a Multi-Omics Approach
Source: Front Microbiol. 2017 Feb 14;8:220. doi: 10.3389/fmicb.2017.00220 (PMC5306329; doi:10.3389/fmicb.2017.00220)
Supplement: Supplementary file 5 [file Table5.DOCX]

**Table S5** Growth rate of *Gramella* strains with monosaccharides and polysaccharide（h^-1^）based OD_600_ measurements (three replicates, ANOVA, p<0.05).

|  | ***G. flava* JLT2011** | ***G. forsetii* KT0803** |
| --- | --- | --- |
| D-glucose | 0.110±0.005 | 0.155±0.005 |
| D-xylose | 0.088±0.007 | - |
| D-galacturonic acid | 0.092±0.002 | 0.070±0.002 |
| L-arabinose | 0.057±0.004 | 0.074±0.004 |
| L-rhamnose | 0.096±0.002 | - |
| D-mannose | 0.095±0.002 | 0.123±0.002 |
| D-galactose | 0.196±0.002 | 0.064±0.005 |
| N-acetyl-D-glucosamine | - | - |
| D-trehalose | 0.075±0.003 | 0.107±0.004 |
| Strach | 0.158±0.005 | 0.120±0.003 |
| Xylan | 0.097±0.007 | - |
| Pectin (from apple) | 0.064±0.005 | 0.029±0.005 |
| Pectin (from citrus) | + | + |
| Galactomannan | + | + |
| Mannan | 0.151±0.007 | - |
| Arabinan | 0.105±0.007 | 0.101±0.007 |
| Arabinogalactan | 0.127±0.006 | 0.061±0.006 |
| Laminarin | 0.088±0.002 | 0.067±0.003 |
| Alginate | - | 0.088±0.032 |
| Chitin | - | - |

“+”, the bacterial growths were visible in media containing citrus pectin or galactomannan, however, it was difficult to determine OD_600_ because of some insoluble particles of citrus pectin and the small volume of a bacterial culture with galactomannan. “-”, no growth.
