# Supplementary material for: Characterization of Potential Polysaccharide Utilization Systems in the Marine Bacteroidetes Gramella Flava JLT2011 Using a Multi-Omics Approach
Source: Front Microbiol. 2017 Feb 14;8:220. doi: 10.3389/fmicb.2017.00220 (PMC5306329; doi:10.3389/fmicb.2017.00220)
Supplement: Supplementary file 9 [file Image1.PDF]

Supplementary Figures

Characterization of Potential Polysaccharide Utilization Systems in the Marine *Bacteroidetes Gramella flava* JLT2011 Using a Multi-omics Approach

Kai Tang*, Yingfan Lin, Yu Han, Nianzhi Jiao*

State Key Laboratory for Marine Environmental Science, Institute of Marine Microbes and Ecospheres, Xiamen University, Xiamen 361102, P. R. China

* Corresponding: tangkai@xmu.edu.cn and jiao@xmu.edu.cn

Other PULs in the Genome of *G. flava* JLT2011

Four *Gramella* strains all contained a starch (or glucan) PUL and a laminarin PUL (Supplementary Figure S3). Both *G. flava* JLT2011 and *G. forsetii* KT0803 can utilize starch and laminarin determined by the growth experiments with these polysaccharides as carbon sources. *G. flava* JLT2011 has no capacity to degrade alginate, whereas *G. forsetii* can degrade it (Supplementary Figure S6). Neither *G. flava* JLT2011 nor *G. echinicola* DSM19838 accommodate the alginate PUL present in *G. forsetii* KT0803 and *G. portivictoriae* DSM23547 (Supplementary Figure S3). Both *G. flava* JLT2011 and *G. forsetii* KT0803 encoded a predicted arabinan-containing glycoside hydrolyase (GH43 and GH51), a susC-susD system and a gene set responsible for L-arabinose utilization (*araBDA*) (Supplementary Figure S4), which were probably involved in their arabinan utilization (Supplementary Figure S6).

Gramella flava JLT2011 harbored a mannan PUL containing mnnA-encoding mannosidases (GH92) and a glycoside hydrolase (GH125) that targets α-1,2- and α-1,6-mannosidic linkages of yeast mannans, respectively (Supplementary Figure S4). A similar gene content of a mannan PUL was previously found in *L. blandensis* MED217 (Supplementary Figure S4). *G. flava* JLT2011 could utilize mannans from yeast (α-1,2-mannans) and *G. forsetii* KT0803 could utilize D-mannose for growth (Supplementary Figure S6), while *G. forsetii* KT0803 could not hydrolyse yeast mannan due to the lack of related GHs. *G. flava* JLT2011 contained other mannan PUL containing genes encoding mannosidase (GH5 and GH26), with activity against the β-1,4-linked mannosides backbone present in plant cell mannans (Supplementary Figure S4). Furthermore, a predicted *susC-susD* system for mannan oligocharrides and a symporter for the degradation product mannose, as well as the transcription regulator *araC*, were present in *G. flava* (Supplementary Figure S4). This suggests that this species has the potential to degrade plant mannan. This PUL exhibits several differences in organization and gene content compared with members of *Bacteroides* (Supplementary Figure S4).

G. flava JLT2011 and *G. forsetii* KT0803 also lacked the capacity to utilize chitin and the degradation product N-acetyl-D-glucosamine (Supplementary Figure S6). Both *G. flava*

JLT2011 and *G. echinicola* DSM19838 contained genes encoding a PUL-like system for chitin (Supplementary Figure S5), where there is a chitinase and N-acetyl-D-glucosamine assimilation pathway without the key gene *nagA* encoding N-acetyl-D-glucosamine 6-phosphate deacetylase (Yadav et al., 2011).

Reference

Yadav, V., Panilaitis, B., Shi, H., Numuta, K., Lee, K., and Kaplan, D. L. (2011). N-acetylglucosamine 6-phosphate deacetylase (nagA) is required for N-acetyl glucosamine assimilation in *Gluconacetobacter xylinus*. *PLoS One* 6, e18099. doi:10.1371/journal.pone.0018099.

Supplementary Tables

Table S1 General features of *Gramella* strains genomes sequenced.

Table S2 Summary and distributions of CAZymes in marine microbes. GH, glycoside hydrolases; CE, carbohydrate esterases; GT, glycosyltransferases; PL, polysaccharide lyases.

Table S3 Genomically predicted glycoside hydrolases (GHs) genes in *G. flava* JLT2011 and their homologs in other strains.

Table S4 Summary and distributions of peptidases in marine microbes.

Table S5 Growth rate of *Gramella* strains with monosaccharides and polysaccharide（h^{-1}）based OD_{600} measurements (ANOVA, $p<0.05$).

Table S6 Comparison of gene expression of *G. flava* JLT2011 growth on xylan and glucose. The up-regulation and down-regulation genes under xylan treatment relative to glucose treatment are shown in red and green, respectively (fold change ≥ 2, or ≤ 0.5; p-value < 0.05). COG, clusters of orthologous groups.

Table S7 Comparison of gene expression of *G. flava* JLT2011 growth on pectin and glucose. The up-regulation and down-regulation genes under pectin treatment relative to glucose treatment are shown in red and green, respectively (fold change > 2, or < 0.5; p-value < 0.05).

Table S8 Functional proteomics analysis of *G. flava* JLT2011 under xylan, pectin and glucose treatments. All detected proteins have at least two unique peptides. +, detected.

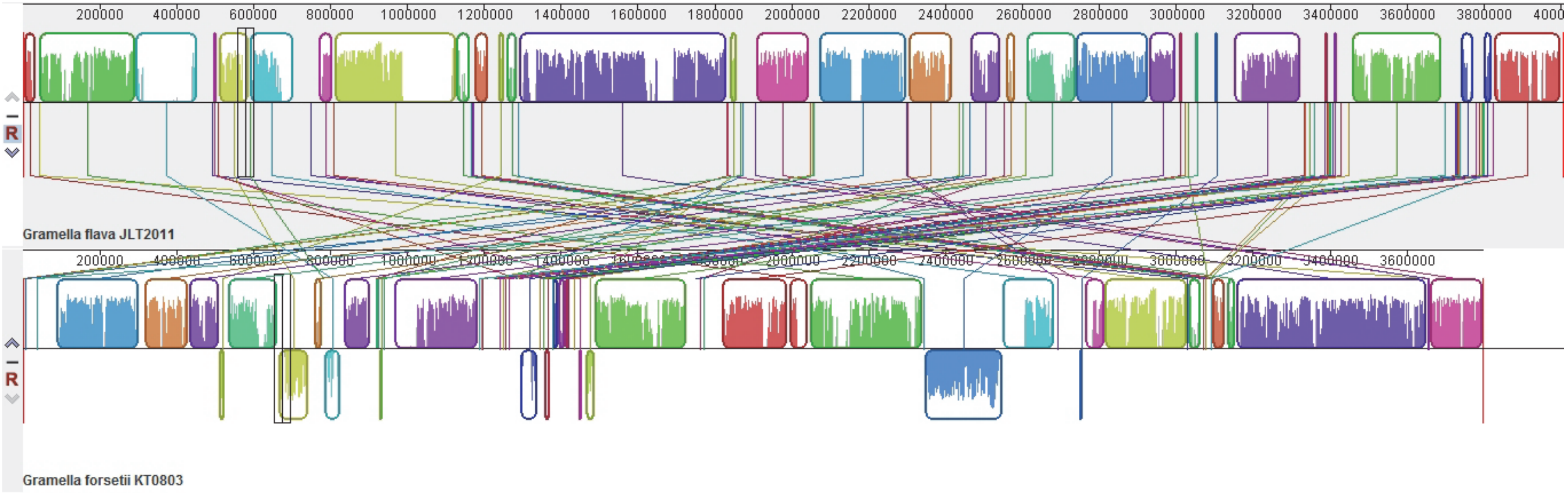

Figure S1. Multiple genome alignment performed using the Mauve progressive software and the chromosomes of *G. flava* JLT2011 and *G. forsetii* KT0803. Lines link blocks where there is homology between two genomes. The colored bars inside the blocks are related to the level of sequence identity. Blocks anchored underneath the horizontal lines indicate regions with inverse orientations.

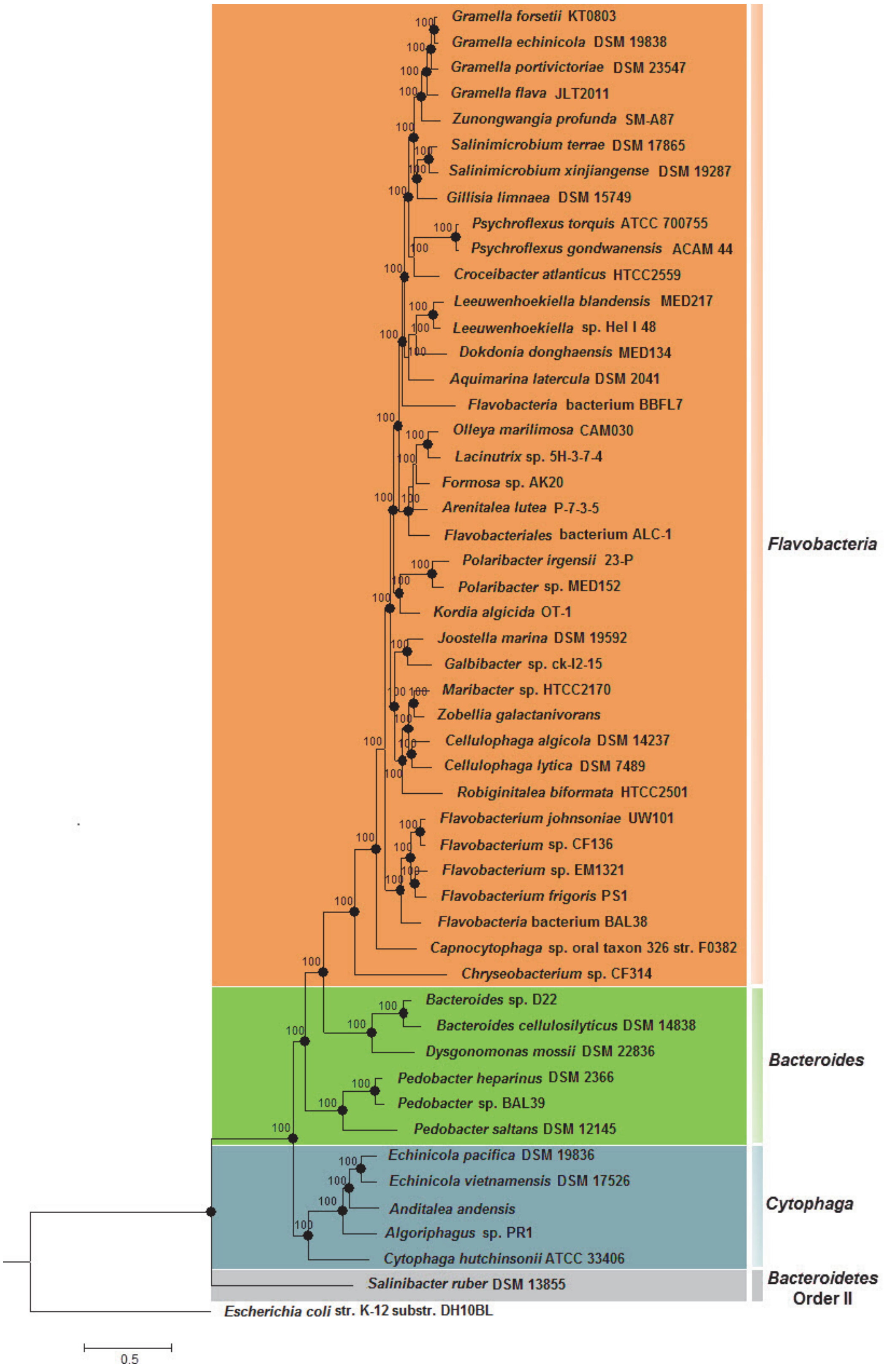

Figure S2. The phylogenetic tree was constructed based on a concatenation of eight

single-copy genes present in all *Bacteroidetes* genomes, including ispU (undecaprenyl pyrophosphate synthase), recR (recombination protein), metG (methionyl-tRNA synthetase), rplS (50S ribosomal protein L19), rplF (50S ribosomal protein L6), rplE (50S ribosomal protein L5), yidC (putative inner membrane protein translocase component) and rpsC (30S ribosomal protein S3). Bootstrap percentages are shown on each branch (1000 replications). Bar = 0.5 substitutions per nucleotide position.

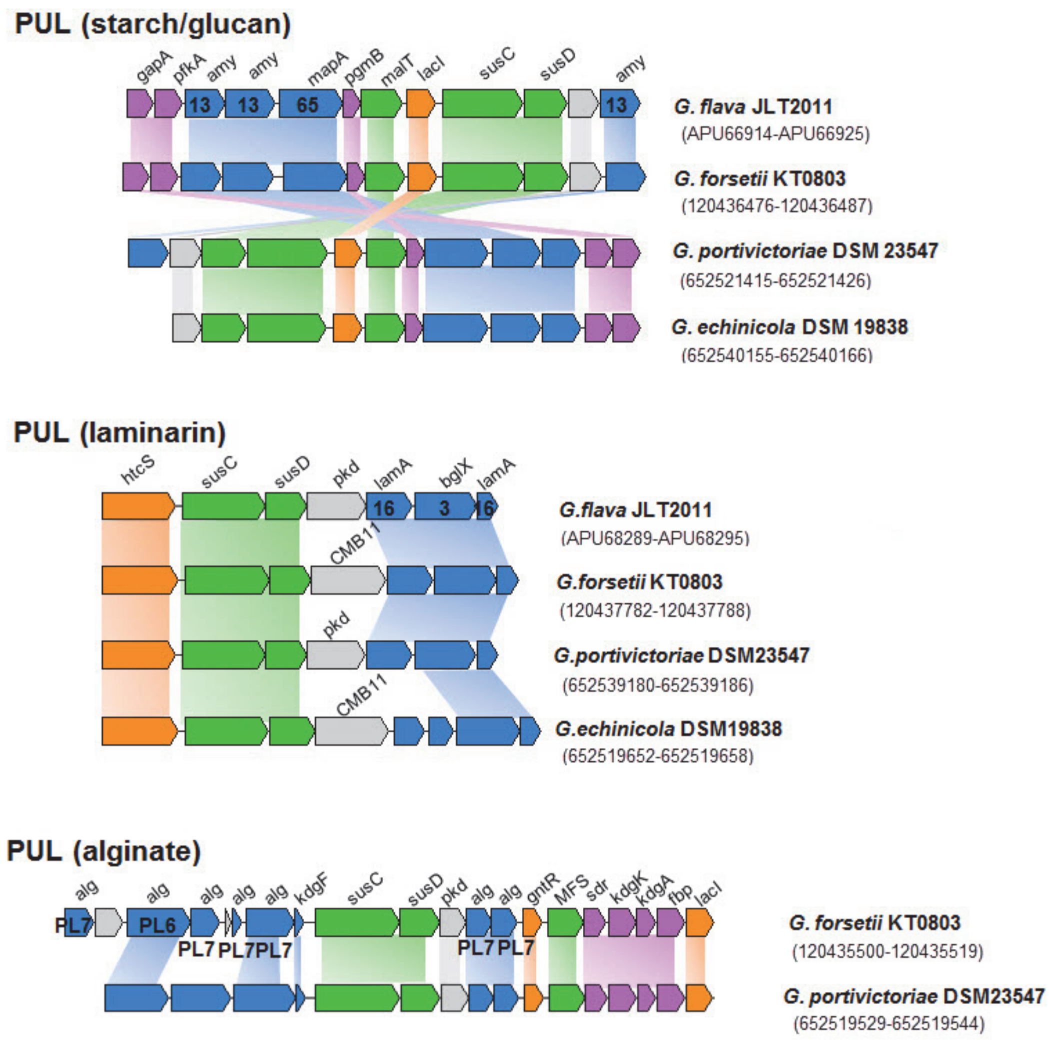

Figure S3. Genetic organization of the starch/glucan, laminarin and alginate PULs in *Gramella* species. The range of Genbank

accession numbers of sequences of the gene cluster in the genome is shown in a bracket. The functions of the proteins are color-coded: blue, CAZymes; green, membrane proteins involved in binding/transport; purple, other enzymes; orange, putative regulation factor; grey, unknown function. Homologous genes are connected by colored bars between two PULs. The CAZymes superfamily classification was listed on a gene. **strach/glucan PUL**: *amy*, Alpha-amylase; *mapA*, Maltose phosphorylase; *pgmB*, Beta-phosphoglucomutase; *gapA*, NAD-dependent glyceraldehyde-3-phosphate dehydrogenase; *pfkA*, 6-phosphofructokinase; *susC*, TonB-dependent receptor; *susD*, Carbohydrate-binding protein; *Malt*, Maltose transporter; *lacI*, LacI family transcriptional regulator; **laminarin PUL:** *lamA*, Laminarinase; *bglX*, Periplasmic beta-glucosidase; *pkd*, PKD Domain-containing protein; *susC*, TonB-dependent receptor; *susD*, Carbohydrate-binding protein; *htcS*, Two-component system sensor histidine kinase/response regulator hybrid; **alginate PUL:** *alg*, Alginate lyase; *pl*, Alginate lyase; *kdgF*, Pectin degradation protein; *pkd*, PKD domain-containing protein; *sdr*, Short-chain dehydrogenase/reductase; *kdgK*, 2-dehydro-3-deoxygluconokinase; *kdgA*, Keto-hydroxyglutarate-aldolase; *fbp*, Fructose-1,6-bisphosphatase; *susC*, TonB-dependent receptor; *susD*, Carbohydrate-binding protein; *MFS*, Hexuronate/hexarate transporter; *gntR*, GntR family transcriptional regulator.

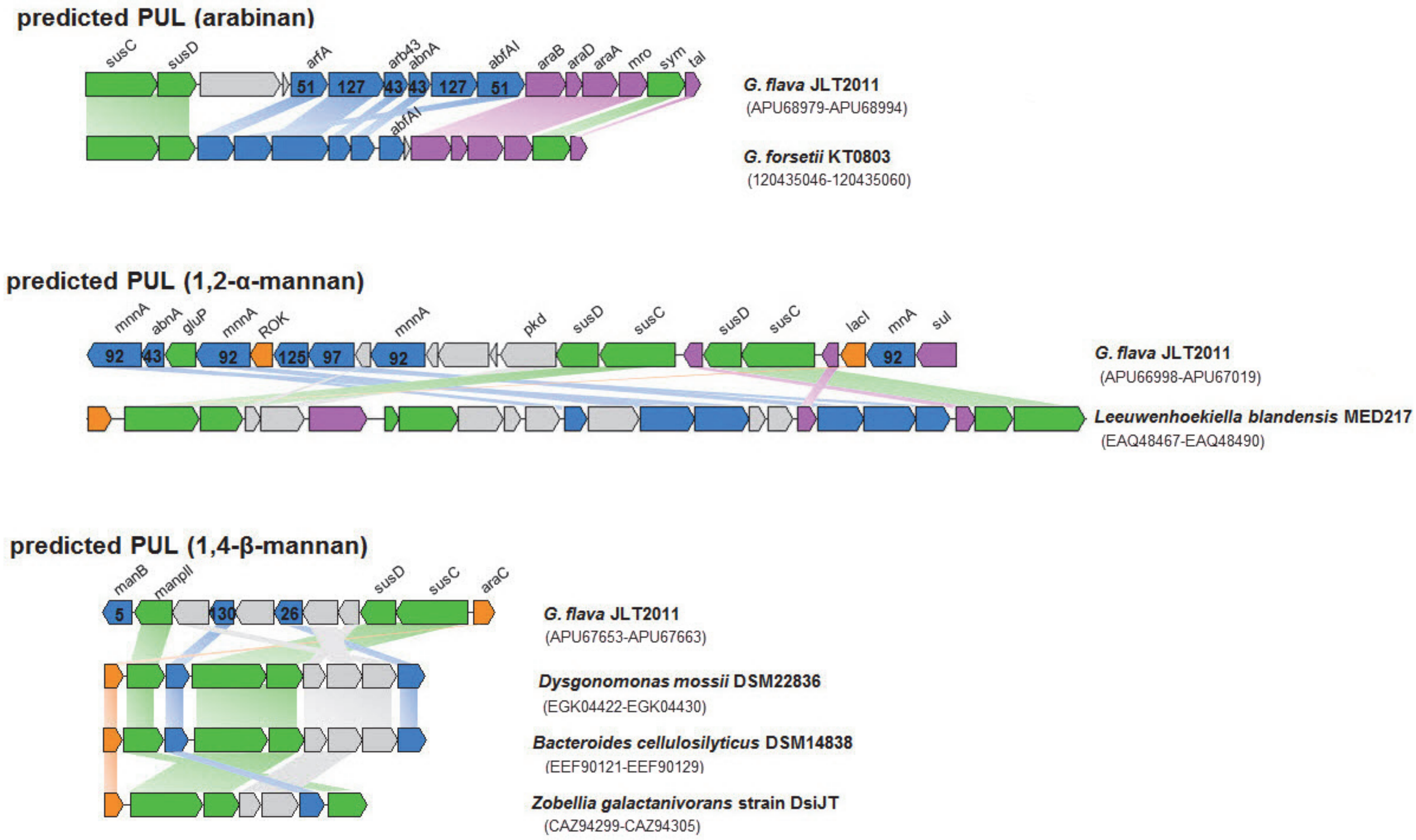

Figure S4. Genetic organization of the predicted PULs of *G. flava* JLT2011. The range of Genbank accession numbers of sequences of the gene cluster in the genome is shown in a bracket. The functions of the proteins are colour-coded: blue, CAZymes;

green, membrane proteins involved in binding/transport; purple, other enzymes; orange, putative regulation factor; grey, unknown function. Homologous genes are connected by colored bars between two PULs. The CAZymes superfamily classification was listed on a gene. **predicted arabinan PUL:** *arfA*, Alpha-N-arabinofuranosidase; *arb43*, Arabinosidase; *abnA*, Arabinan endo-1,5-alpha-L-arabinosidase; *abfAI*, Alpha-N-arabinofuranosidase; *araB*, Ribulokinase; *araD*, L-ribulose-5-phosphate 4-epimerase; *araA*, L-arabinose isomerase; *mro*, Aldose 1-epimerase; *sym*, Sodium:solute symporter family protein; *tal*, Transaldolase; **predicted 1,2-α-mannan PUL:** *mnnA*, Alpha-1,2/3-mannosidase; *abnA*, Arabinan endo-1,5-alpha-L-arabinosidase; GH125, alpha-1,6-mannanase; *susB*, Alpha-glucosidase; *pkd*, PKD domain-containing protein; *sul*, Arylsulfatase; *susC*, TonB-dependent receptor; *susD*, Carbohydrate-binding protein; *ROK*, ROK family member transcriptional repressor; *lacI*, LacI family transcriptional regulator; **predicted 1,4-β-mannan PUL:** *manB*, Endo-1,4-beta-mannosidase; *manpII*, mannose Sodium solute transporter; *araC*, AraC family transcriptional regulator.

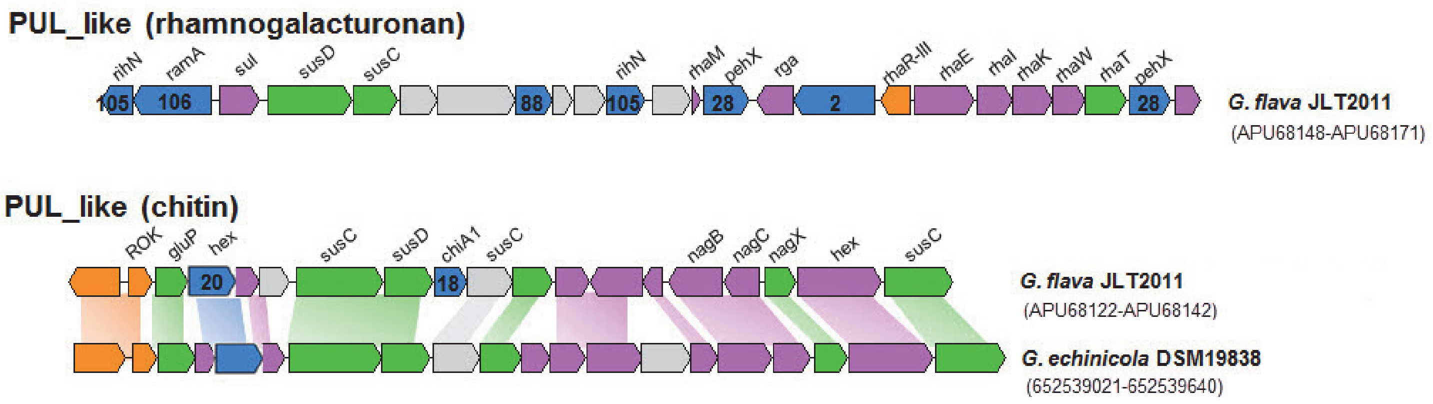

Figure S5. Genetic organization of the two PUL-like operons of *G. flava* JLT2011. The range of Genbank accession numbers of sequences of the gene cluster in the genome is shown in a bracket. The functions of the proteins are color-coded: blue, CAZymes; green, membrane proteins involved in binding/transport; purple, other enzymes; orange, putative regulation factor; grey, unknown function. Homologous genes are connected by colored bars between two PULs. The CAZymes superfamily classification was listed on a gene. **rhamnogalacturonan PUL_like:** *pehX*, Exo-polygalacturonosidase; *rihN*, Rhamnogalacturonides degradation protein; *rga*, Rhamnogalacturonan acetylesterase; *ramA*, Alpha-L-rhamnosidase; *rhaM*, L-rhamnose mutarotase; *rhaE*, Predicted rhamnulose-1-phosphate aldolase; *rhaI*, L-rhamnose isomerase; *rhaK*, Rhamnulokinase; *rhaW*, L-lactate dehydrogenase; *rhaT*, Pedicted L-rhamnose permease; *susC*, TonB-dependent receptor; *susD*, Carbohydrate-binding protein; *rhaR-III*, Transcriptional regulator of rhamnose utilization; **chitin PUL_like:** *chiA1*, Chitinase; *hex*, Beta-hexosaminidase; *nagB*, Glucosamine-6-phosphate deaminase; *nagC*, transcriptional repressor protein; *nagX*, N-acetylglucosamine related transporter; *ROK*, ROK family member transcriptional repressor; *susC*, TonB-dependent receptor; susD, Carbohydrate-binding protein.

Xylan

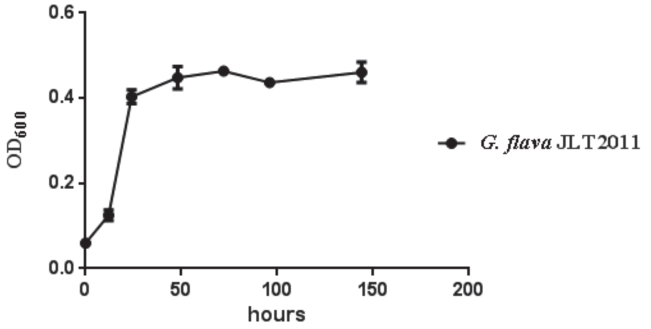

D-xylose

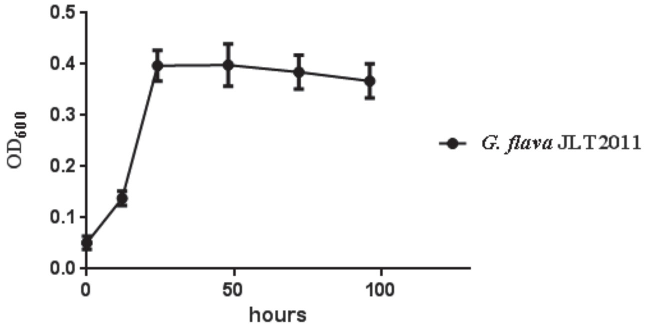

Pectin (from apple)

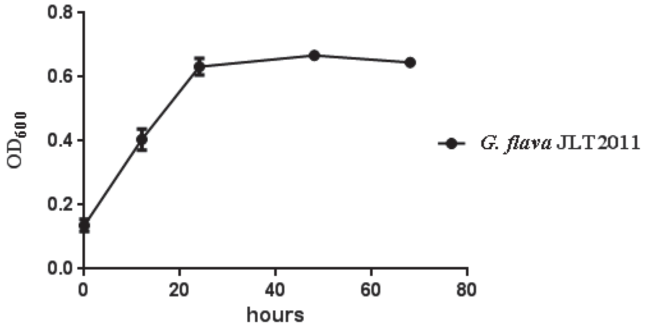

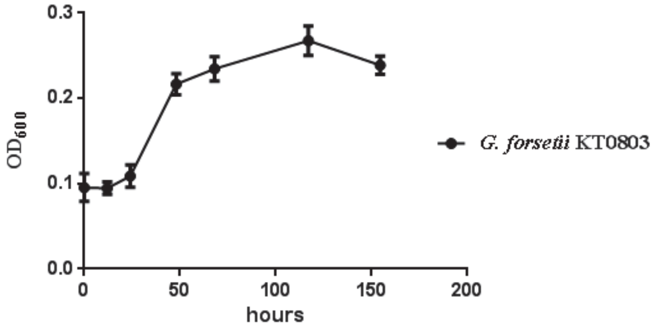

D-galacturonic acid

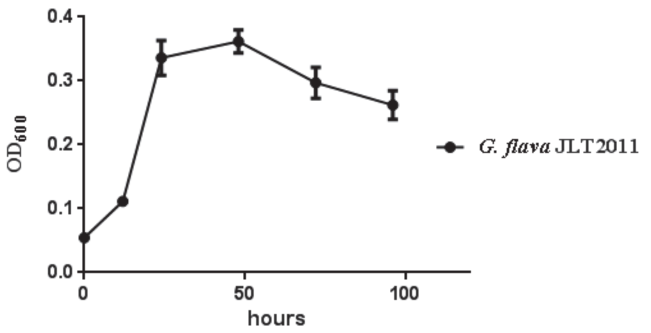

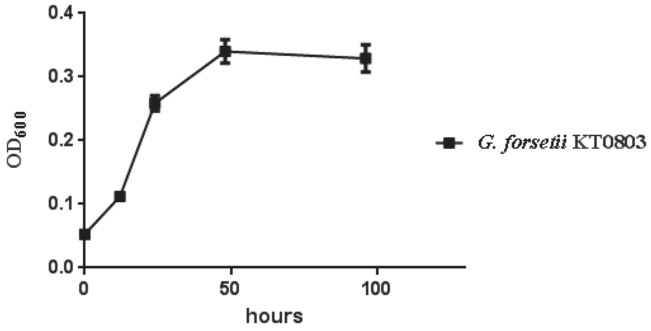

D-glucose

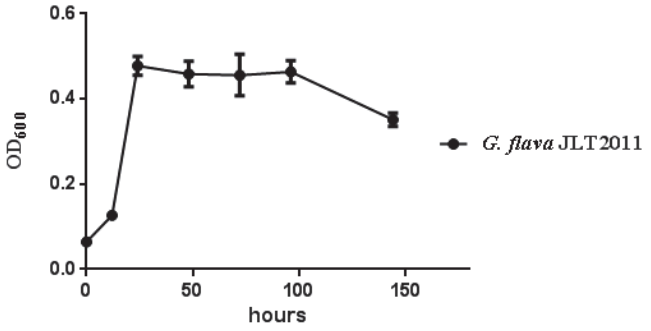

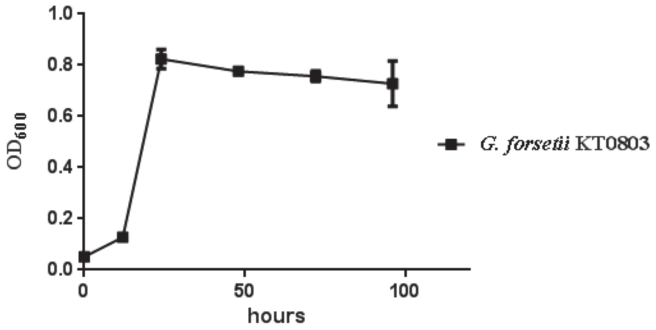

L-arabinose

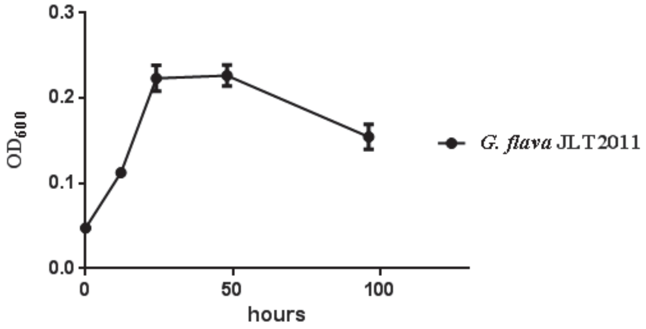

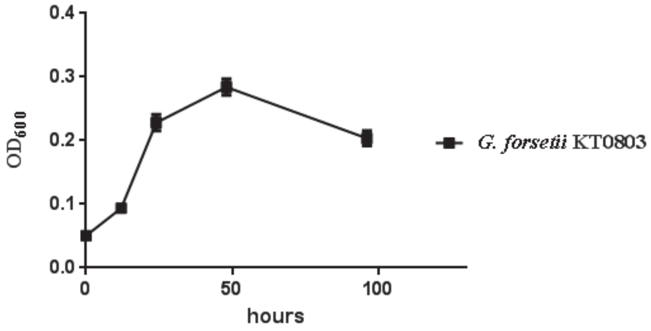

L-rhamnose

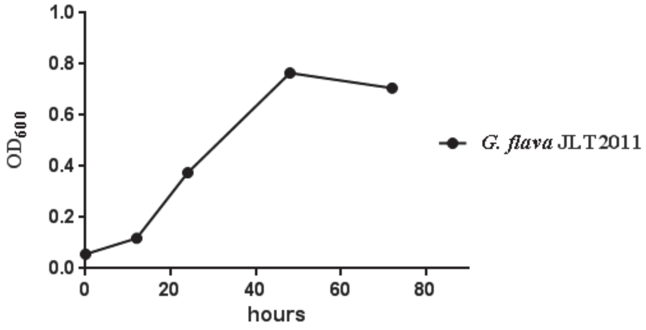

D-mannose

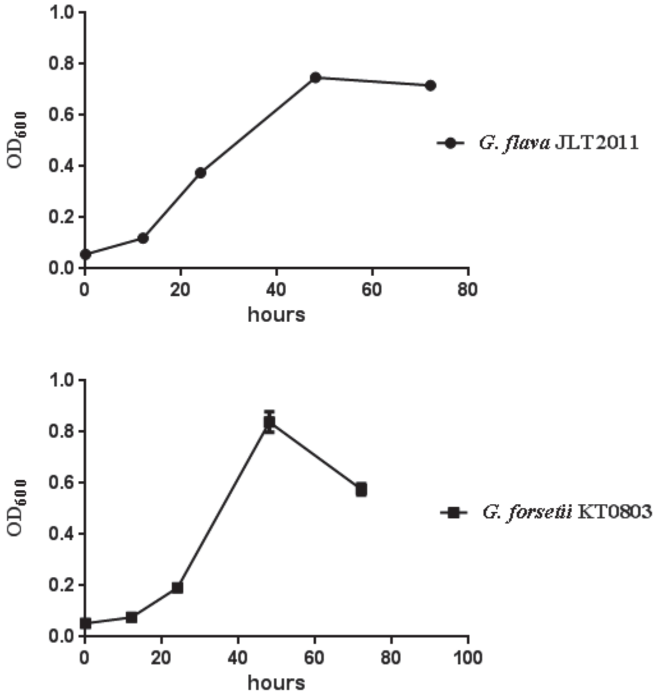

D-galactose

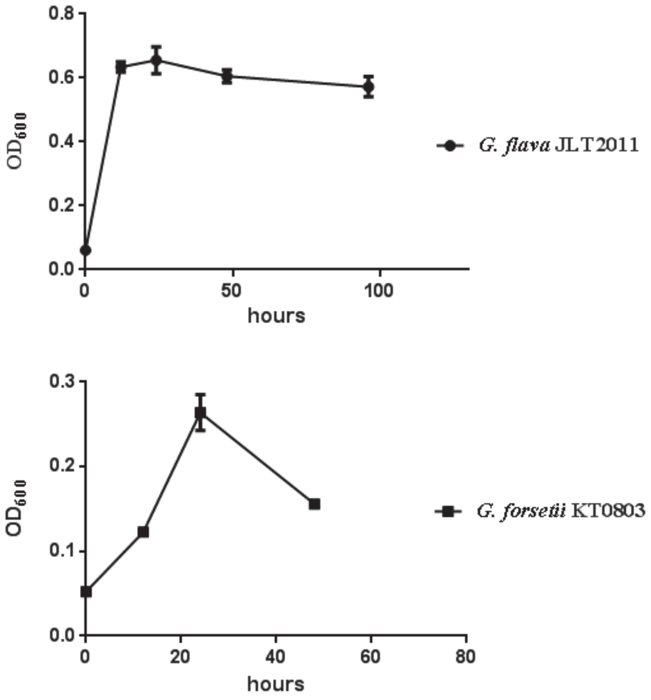

D-trehalose

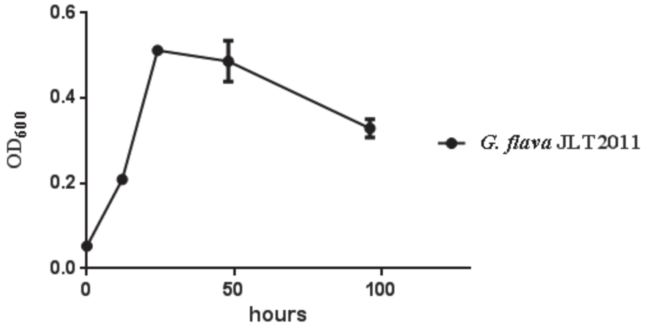

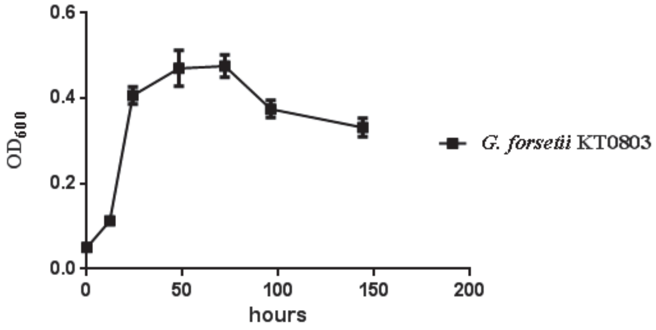

Strach

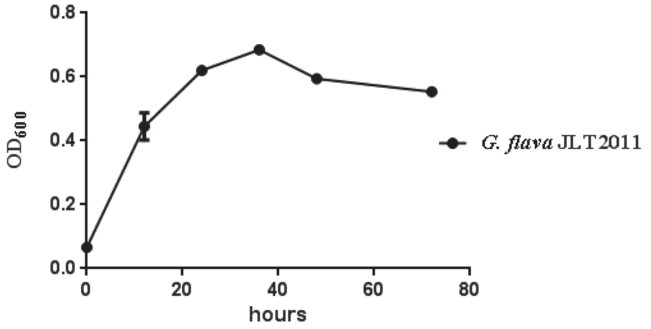

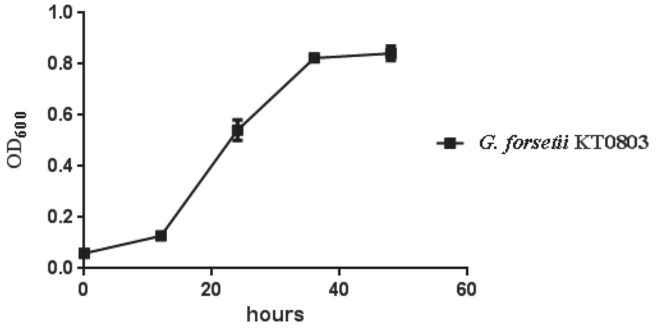

Mannan

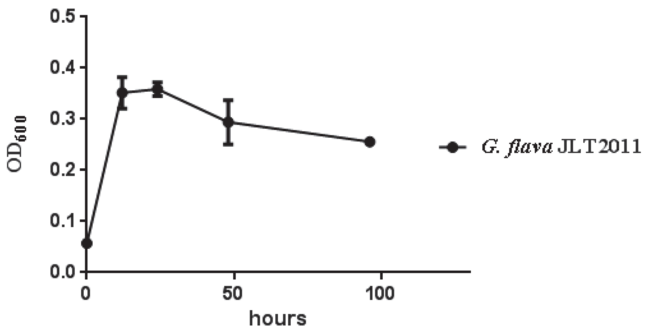

Arabinan

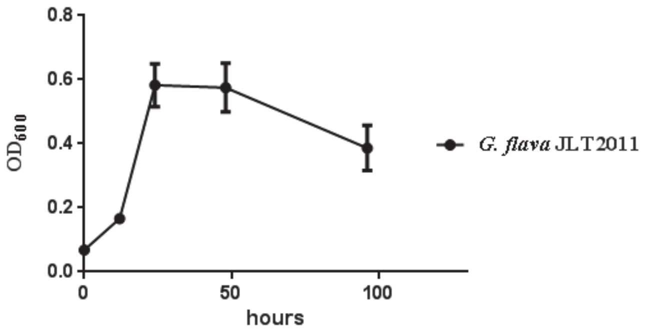

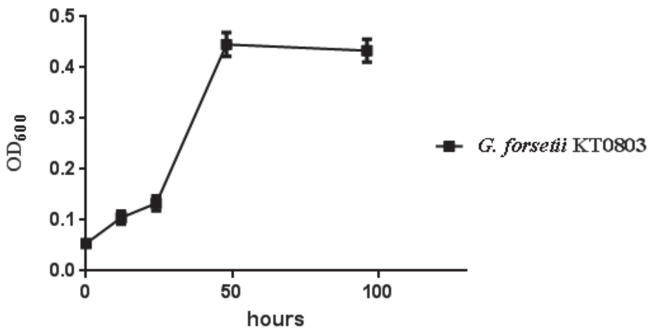

Arabinogalactan

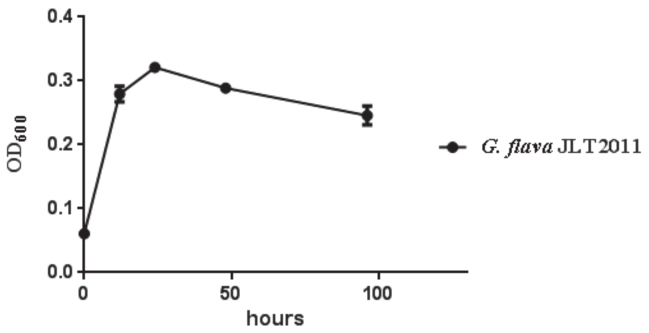

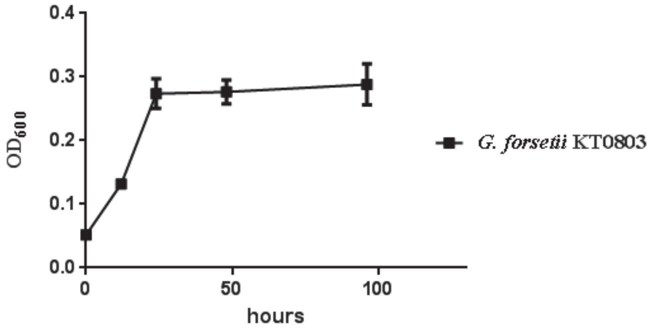

Laminarin

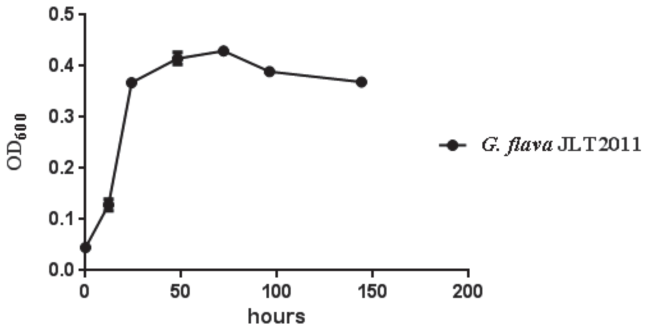

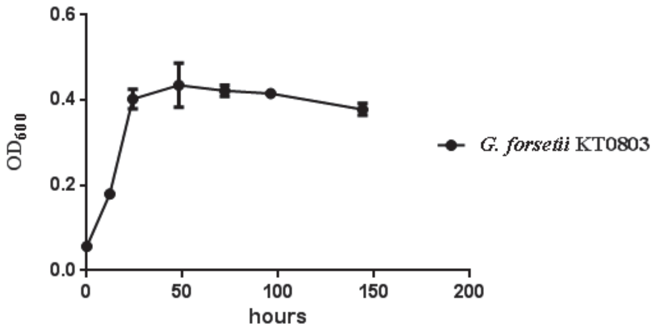

Alginate

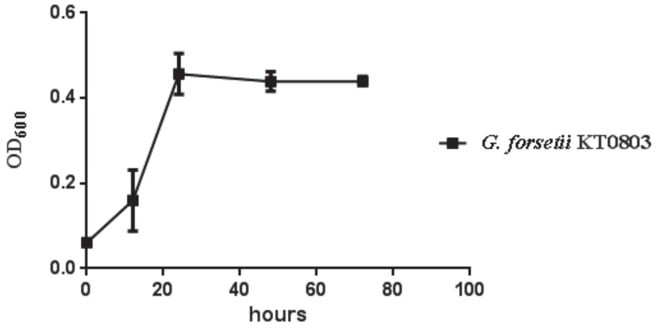

Figure S6. Growth curves of *Gramella* strains with monosaccharides and polysaccharide

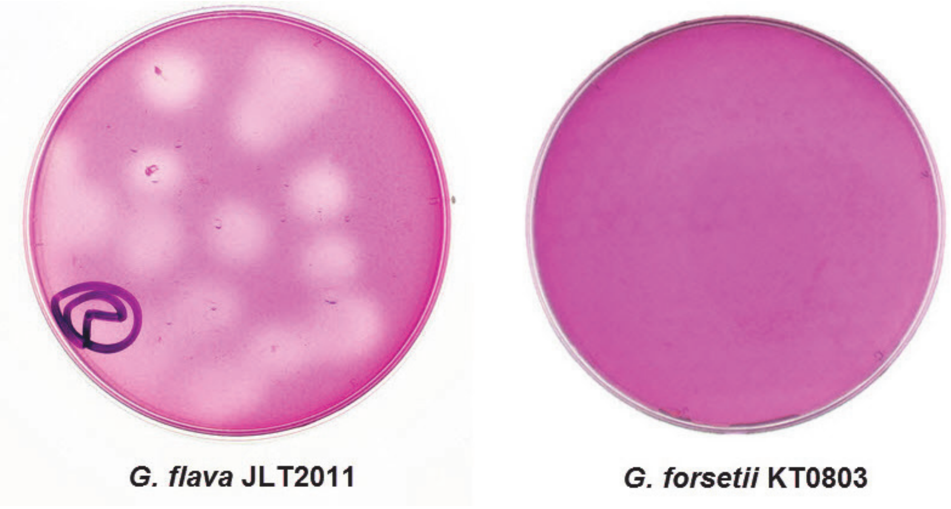

Figure S7. The pectin methylesterase activity of *Gramella* species measured by ruthenium red staining method. Only *G. flava* JLT2011 showed pectin methylesterase activity in apple pectin.
